# Fluorescently labeled xylosides offer insight into the biosynthetic pathways of glycosaminoglycans†

**DOI:** 10.1039/d1ra06320k

**Published:** 2021-11-29

**Authors:** Roberto Mastio, Daniel Willén, Zackarias Söderlund, Gunilla Westergren-Thorsson, Sophie Manner, Emil Tykesson, Ulf Ellervik

**Affiliations:** Centre for Analysis and Synthesis, Centre for Chemistry and Chemical Engineering, Lund University P. O. Box 124 SE-221 00 Lund Sweden ulf.ellervik@chem.lu.se; Department of Experimental Medical Science, Lund University P. O. Box 117 SE-221 00 Lund Sweden

## Abstract

Five novel xylosides tagged with the fluorescent probe Pacific Blue™ were synthesized and found to act as substrates for β4GalT7, a bottleneck enzyme in the biosynthetic pathways leading to glycosaminoglycans. By confocal microscopy of A549 cells, we showed that the xylosides were taken up by the cells, but did not enter the Golgi apparatus where most of the glycosaminoglycan biosynthesis occurs. Instead, after a possible double galactosylation by β4GalT7 and β3GalT6, the biosynthesis was terminated. We hypothesize this is due to the charge of the fluorescent probe, which is required for fluorescent ability and stability under physiological conditions.

## Abstract 2

Fluorescently labeled xylosides are taken up by cells and initiate priming of labeled GAG chains of various length.

## Graphical abstract

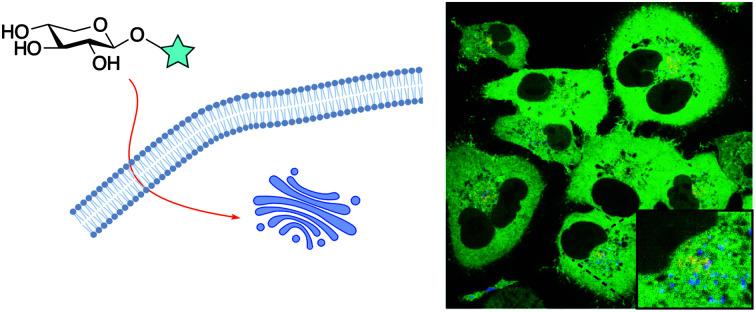

## Introduction

Proteoglycans (PG) are macromolecules that consist of long, negatively charged, linear carbohydrate chains, *i.e.* glycosaminoglycans (GAGs), attached to the surface of a core protein. Proteoglycans are mainly located in the extracellular matrix of mammalian cells and are involved in processes such as tissue development, cellular growth, adhesion, and coagulation.^1^ The GAG chains are composed of alternating disaccharides of (-4)GlcNAc(β1-4)GlcA(β1-) for heparan sulfate (HS) or (−3)GalNAc(β1-4)GlcA(β1-) for chondroitin sulfate (CS). The growing polymer is concomitantly modified by epimerization of GlcA to IdoA, to form dermatan sulfate (DS) from CS, and sulfation of both the uronic acids and the hexosamines, results in extensive structural diversity. The enzymes of the biosynthetic pathway of GAG synthesis are known and, in many cases, cloned and expressed. However, the organization of these enzymes in the Golgi and ER is known to a much less extent. According to the GAGosome theory, some of these enzymes are co-localized and work synchronously.^2^

**Fig. 1:**
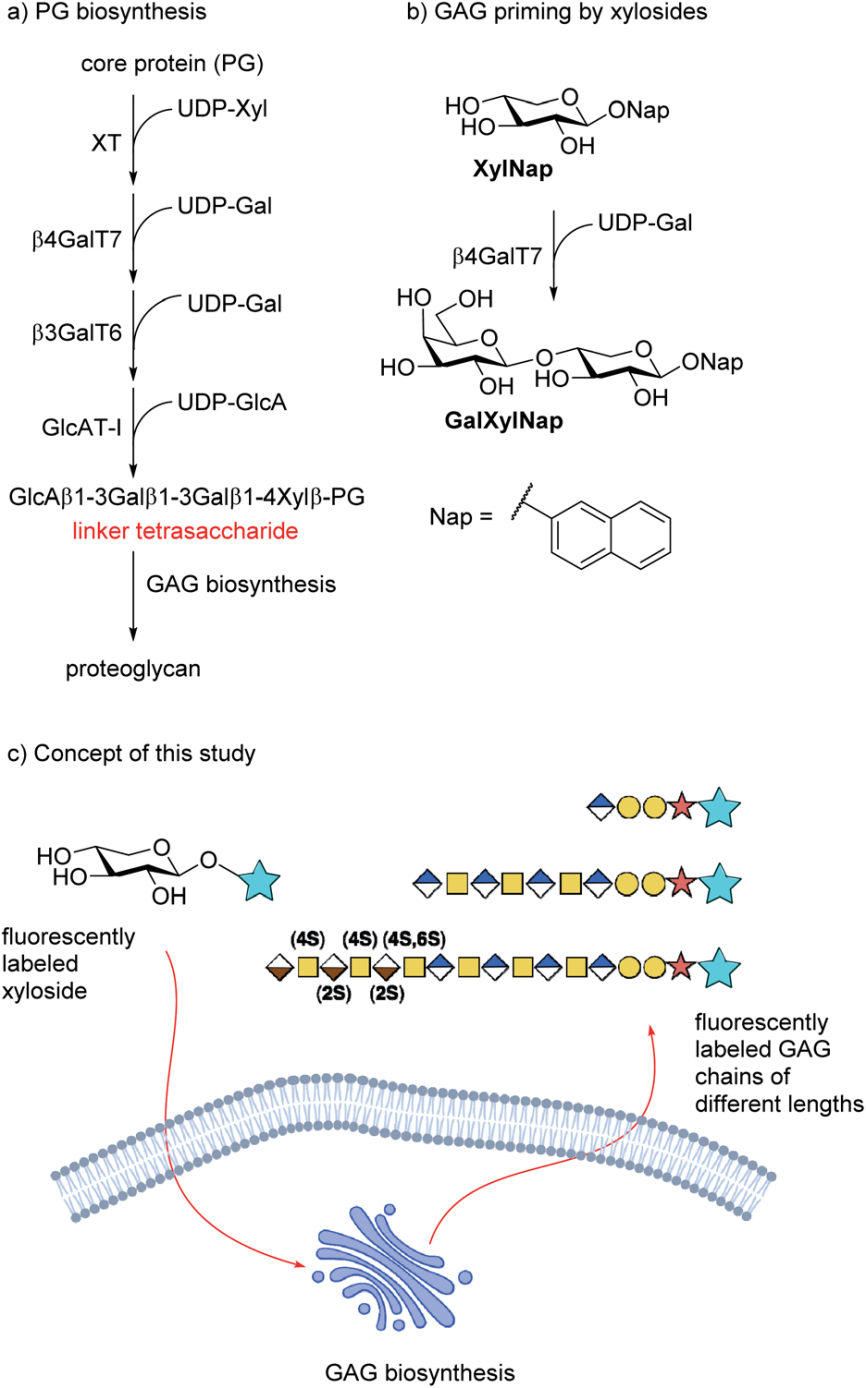
(a) Biosynthesis of the linker tetrasaccharide of HS and CS/DS. (b) Priming of GAG synthesis by xylosides is initiated by galactosylation of XylNap by β4GalT7 to form GalXylNap. (c) Concept of this study. Fluorescently labeled xylosides are expected to be taken up by cells and initiate GAG priming to release fluorescently labeled GAG chains.

The disaccharide polymer is bridged by a linker region, which for HS and CS/DS consists of a tetrasaccharide motif, *i.e.* GlcA(β1-3)Gal(β1-3)Gal(β1-4)Xylβ (*cf.*Fig. 1a). The formation of these GAGs is initiated by xylosylation of a serine residue in the core protein followed by galactosylation of the xyloside moiety by β-1,4-galactosyltransferase 7 (β4GalT7). Interestingly, xylose is a rather unusual carbohydrate in mammalian cells and thus a potential selective target for intervention of the GAG biosynthesis pathway.

It has been known since the 1970s that β-d-xylopyranosides with hydrophobic aglycons can permeate cell membranes and initiate GAG synthesis in competition with the natural PG synthesis (Fig. 1b). Since these GAGs are not connected to a protein core, they are soluble and usually secreted into the extracellular space. The amounts and structure of the primed GAGs are dependent on both aglycon and cell type.^2–11^

We have earlier presented xylosides with aglycons based on a naphthalene core (*e.g.* naphthyl β-d-xylopyranoside, XylNap, *cf.*Fig. 1b) and we have investigated the effects of different xylosides on the GAG fine structure.^12–15^

The enzymatic mechanism for the first galactosylation step was clarified by cloning and expression of β4GalT7 by Almeida *et al.* in 1999.^16^ Later on, the crystal structure of human β4GalT7 was resolved by Qasba and co-workers in 2013 and showed that a conformational change upon binding of UDP or UDP-Gal formed a hydrophobic, xyloside binding, pocket.^17^ The galactosylation then proceeds with an S_N^2^_-like mechanism where the 4-OH of xylose attacks the anomeric position in UDP-Gal with UDP as leaving group. In 2014 we presented a β4GalT7-assay and initiated the search for GAG primers.^18^

To study the biosynthesis, and search for a GAGosome, a fluorescently labeled xyloside that functions as a GAG primer would be highly valuable.^19–21^ For this approach to be successful, the xyloside must be able to first permeate through the cell membrane and then be able to initiate GAG-synthesis.

**Chart 1:**
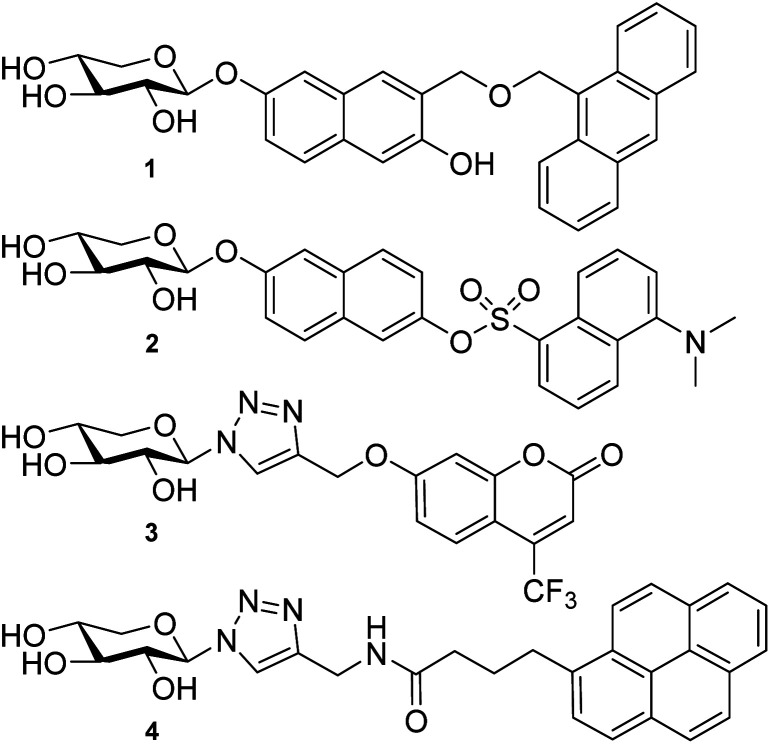
Examples of earlier attempts to fluorescently labeled GAG-priming xylosides.

Previous attempts to design fluorescently labeled xylosides are exemplified in Chart 1. Johnsson *et al.* presented naphthoxylosides fused with anthracene and dansyl groups (1 and 2). These compounds were taken up by the cells but no induction of GAG synthesis could be shown.^11,22^ This was then believed to be due to the bulkiness of the dansyl and the (9-anthracenyl)methyl aglycons. Later, Tran *et al.* synthesized xylosides connected to dansyl and fluorescein groups *via* a triazolyl linkage. Similar to the results of Johnsson *et al.*, these compounds were not able to initiate priming. However, Tran *et al.* did observe GAG synthesis using xylosides carrying umbelliferyl and pyrene groups coupled *via* a triazolyl linkage (3 and 4).^23^ While the pyrene analogs could not be detected by fluorescence, the corresponding umbelliferyl analogs were detected and showed longer HS chains compared to the 4-methylumbelliferyl xylosides not carrying a triazolyl linkage. However, since their quantum yields decrease significantly in water, these conjugates are still not optimal for investigations of the GAG biosynthesis.^24,25^

**Chart 2:**
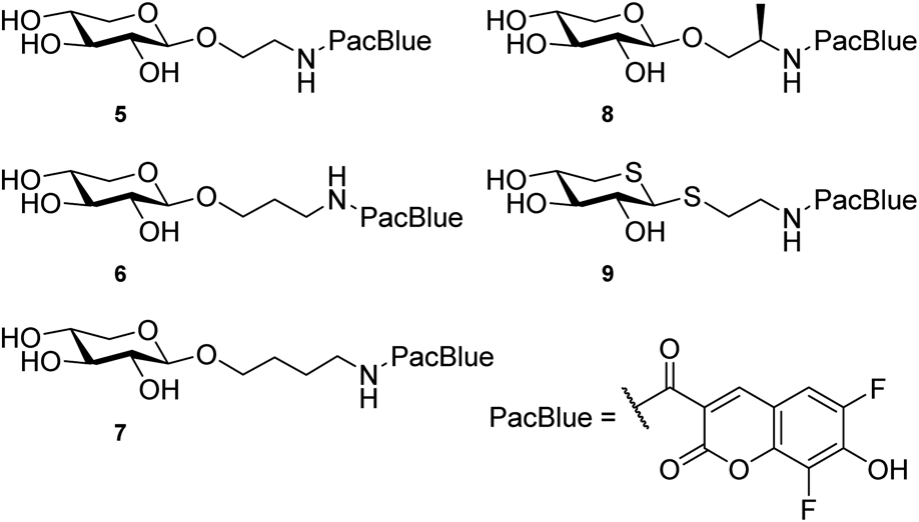
. Structures of the investigated xyloside analogs.

A coumarin-based fluorophore that has gained popularity in cell biology, due to its small size and high quantum yield, is the 6,8-difluoro-7-hydroxycoumarin-3-carboxylic acid, or Pacific Blue™ (referred to as PacBlue in this article, *cf.*Chart 2).^26–29^ The two fluorine atoms flanking the hydroxyl lowers the p*K*_a_ (*i.e.* 3.7, compared to 7.5 of the nonfluorinated coumarin) and it is thus deprotonated at physiological pH, which is important for the fluorescence.^30^ Furthermore, synthetic pathways to the coumarin dye have been published^31,32^ enabling multigram synthesis.^33^ PacBlue can also be used for FRET (Förster Resonance Energy Transfer) studies with other fluorophores, such as the amino acid tryptophan, enabling studies of ligand–protein interaction in proteins with suitably positioned tryptophan residues within the protein scaffold.^32^

We hypothesize that xylosides carrying the PacBlue moiety will be taken up by cells, initiate the GAG biosynthesis and release fluorescently labeled GAGs (Fig. 1c). The ability of PacBlue xylosides to act as a substrate for β4GalT7 has not been tested before, although other coumarin-containing xylosides (*e.g.* methylumbelliferyl) have been shown to be both inhibitors and substrates for GAG-biosynthesis.^34,35^ The rationale of this paper is to explore the binding efficacy of the xylosides carrying the PacBlue moiety (Chart 2) in the β4GalT7 assay,^18^ as well as to determine cellular uptake and GAG priming ability.

## Results and discussion

### Synthesis of PacBlue xylosides

Previous results from us and others have shown that the length of the linker between the xylose moiety and the aglycon significantly influences galactosylation by β4GalT7.^2,21^ Therefore, we decided to synthesize a series of PacBlue xylosides 5–9 (Chart 2) with different linker lengths as well as a dithioxyloside. The later analog has been shown to be a good substrate for β4GalT7.^19^

**Scheme 1:**
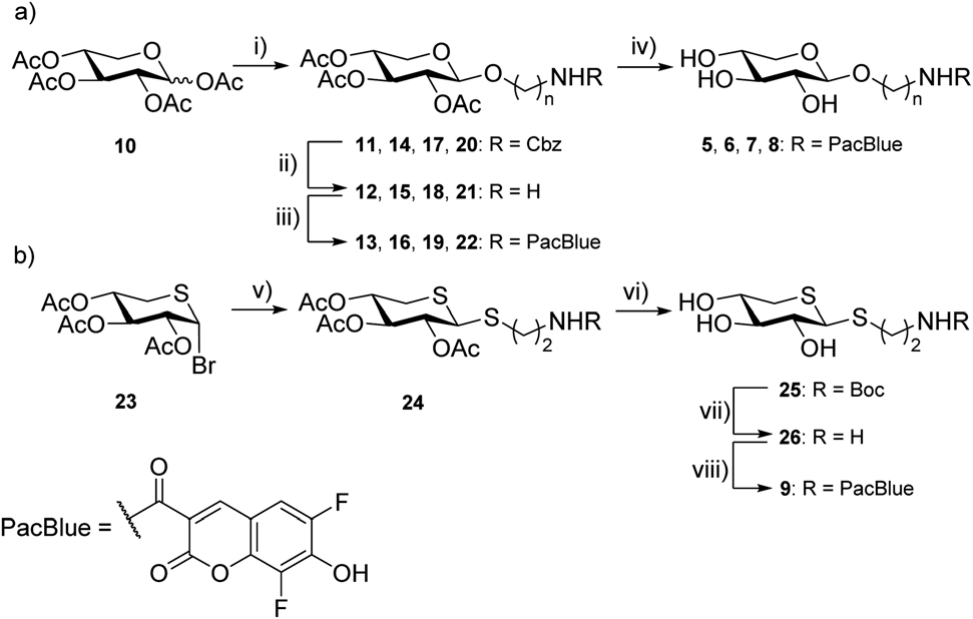
Reagents and conditions (a) (i) Cbz-protected linker, BF_3_·OEt_2_, MeCN, MS 3 Å, 2 h, r.t., 14–21%; (ii) Pd/C, H_2_, MeOH, 2 h; r.t., 62–100%; (iii) Pacific Blue™ carboxylic acid, EDC, HOBt, TEA, DCM, 2 h; r.t., 36–58%; (iv) K_2_CO_3_, MeOH, 1 h; r.t. 96–98%. (b) (v) Boc-protected thiol linker, ZnO–ZnCl_2_, toluene/MeCN (1 : 1), MS 3 Å, 60 °C, 2 h; 40%, β/α 2 : 1; (vi) HCl (1 M), EtOH, 1 h, r.t, 52%; (vii) MeOH, LiOH, 40 min, r.t., 98%, β/α 2 : 1; (viii) Pacific Blue™ succinimidyl ester, DIPEA, DMF, 4 h; r.t, 46%.

Synthesis of targets 5–8 was performed using conventional carbohydrate synthetic methods (Scheme 1a), starting from per-acetylated d-xylose (10) and commercially available, suitably protected linkers. Glycosylation using BF_3_·OEt_2_ in MeCN, with Cbz-protected amino alcohols furbished compounds 11, 14, 17, and 20.

Removal of the Cbz protecting group was performed by hydrogenation (H_2_, Pd/C), to give 12, 15, 18, and 21 as the free amines. These intermediates were then subjected to EDC-promoted coupling using HOBt as a catalyst, furbishing 13, 16, 19, and 22. Final deprotection using K_2_CO_3_ in MeOH gave compounds 5, 6, 7, and 8.

Synthesis of compound 9 required a different strategy (Scheme 1b). Glycosylation was first attempted using the peracetylated thioxyloside in a similar fashion as the other targets with BF_3_·OEt_2_ as the promoter, without success. Previous glycosylations using this donor,^19,36^ have involved aromatic, rather than aliphatic, thiols as acceptors, which could explain the low reactivity. Instead, we used the 2,3,4-tri-*O*-acetyl-5-thio-α-d-xylopyranosyl bromide^37^ (23), and ZnO–ZnCl_2_ as the promoter system in a MeCN-toluene mixture^38^ along with a Boc-protected linker. Indeed, the ZnO–ZnCl_2_ promoter at 60 °C yielded a sufficient amount of 24 as a 2 : 1 β/α-mixture. Deacetylation was performed using LiOH in MeOH and Boc deprotection using HCl in EtOH. Amide coupling was performed using the commercial amine-reactive succinimidyl ester of PacBlue to finally yield 9.

### Measurement of lipophilicity

The partition coefficients of compounds 5–9, in reference to XylNap,^10^ were estimated by gradient HPLC retention times (Table 1).^39,40^ The dithio-analog 9 showed the most similar lipophilicity compared to XylNap.

### Galactosylation by β4GalT7

**Fig. 2:**
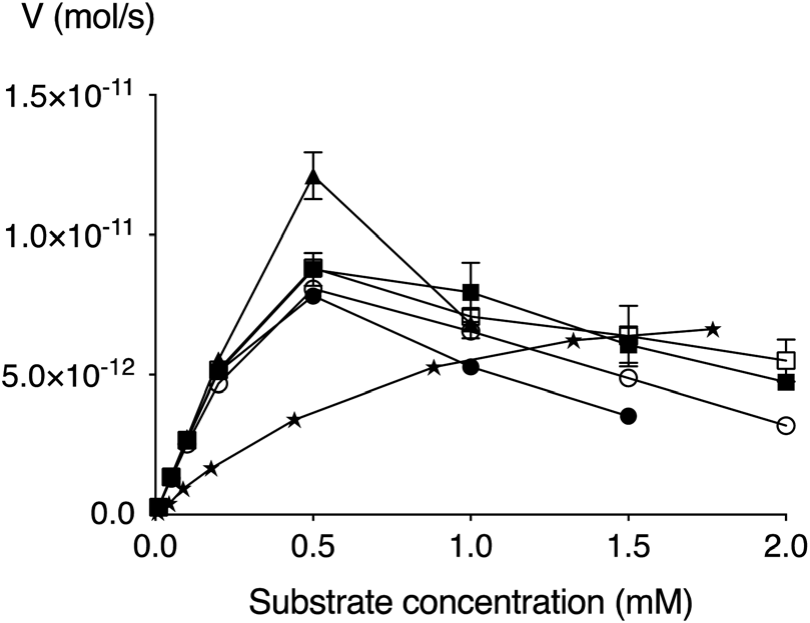
Michaelis–Menten representation of the activity of β4GalT7 (V) as a function of the concentration of XylNap (stars), 5 (solid circle), 6 (open circle), 7 (solid square), 8 (open square), and 9 (triangle).

We have earlier developed an improved enzymatic assay of β4GalT7 substrate activity and/or inhibition.^41^ Compounds 5–9 were thus evaluated using this assay in regards to galactosylation. All compounds were found to be efficiently galactosylated by β4GalT7 up to a concentration of approximately 0.5 mM, after which a substantial amount of substrate inhibition was observed (Fig. 2 and Table 2). Both the *V*_max_ (approximately 0.1 pmol s^−1^) and *K*_m_ (0.1–0.2 mM) of the XylPacBlue derivatives were significantly higher and lower, respectively, compared to XylNap. The sulfur analog (9) had the fastest kinetics, but no big differences were observed between linker length variants (5–7), nor of the branched-chain variant (8).

**Table 1:** Retention times of 5–9 compared to XylNap

Compound	Retention time^a^ (min)
5	9.65 ± 0.01
6	11.7 ± 0.01
7	13.8 ± 0.01
8	13.2 ± 0.01
9	15.5 ± 0.04
XylNap	14.9 ± 0.00

a Gradient from 5 : 95 → 100 : 0 MeCN : water, 1.2% MeCN increase per minute. Retention times are a mean of three measurements.

**Table 2:** Galactosylation of xylosides by β4GalT7

Compound	*K*_m_, (mM)	*V*_max_, (pmol s^−1^)	*k*_cat_, (s^−1^)	*k*_cat_/*K*_m_, (mM^−1^ s^−1^)
5	0.39	14.1	4.4	11.3
6	0.55	17.0	5.3	9.7
7	0.57	18.9	5.9	10.3
8	0.57	19.0	6.0	10.4
9	2.2	65.8	20.6	9.3
XylNap	0.84	10.0	3.1	3.7

We have previously shown that compounds 1 and 2 fail to induce GAG synthesis in human T24 cells. In agreement with those results, we did not observe any galactosylation of these compounds in the β4GalT7 assay (data not shown).

### Uptake, priming, and cellular distribution

In order to be used as tools for studying the GAG biosynthesis, we evaluated the ability of the compounds to be taken up and to initiate biosynthesis of GAGs in living cells. Therefore, compounds 5, 7, and 9, and the acetylated form of 7 (*i.e.* compound 19) were investigated *in vitro* using A549 cells. Acetylated carbohydrates have a significant increase in uptake (up to three orders of magnitude),^42^ and acetylation is a common way of making drugs more permeable across the lipid bilayer, whereupon uptake the esters are cleaved off by nonspecific esterases and reveal the active substrates.^43^

**Fig. 3:**
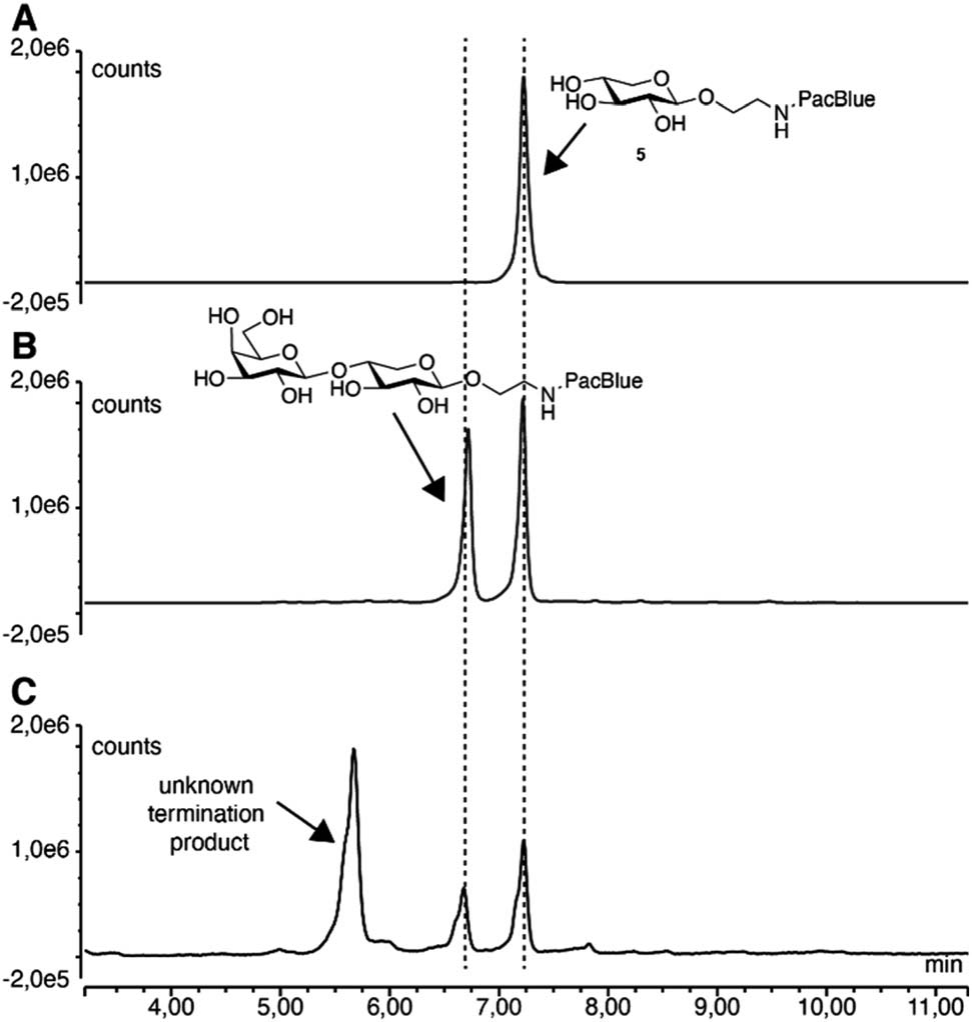
Reversed-phase chromatography analysis of 5 before (A) and after (B) enzymatic treatment, as well as of ion exchange-purified medium from A549 cells treated with 0.1 mM 5 for 24 h (C).

No priming of full-length GAGs was observed for either of the compounds (ESI Fig. 1†). However, when ion exchange-purified cell medium was analyzed by fluorescence-coupled reversed-phase chromatography, we observed secreted products corresponding to Gal-XylPacBlue, confirmed by its chromatographic overlap with the β4GalT7 assay product. Furthermore, we observed slightly larger products possibly corresponding to double galactosylation by β4GalT7 and β3GalT6 (Fig. 3).

The acetylated compound 19 was thus efficiently taken up by the cells and was *in vivo*-deacetylated to form the active compound, but no increase in chain length of secreted products was observed beyond the non-acetylated variant (ESI Fig. 2†). By analyzing cell lysate from A549 cells treated with compound 5, we noticed the same product pattern as in the cell medium and could confirm that there was no accumulation of GAGs within the cells (ESI Fig. 3†).

**Fig. 4:**
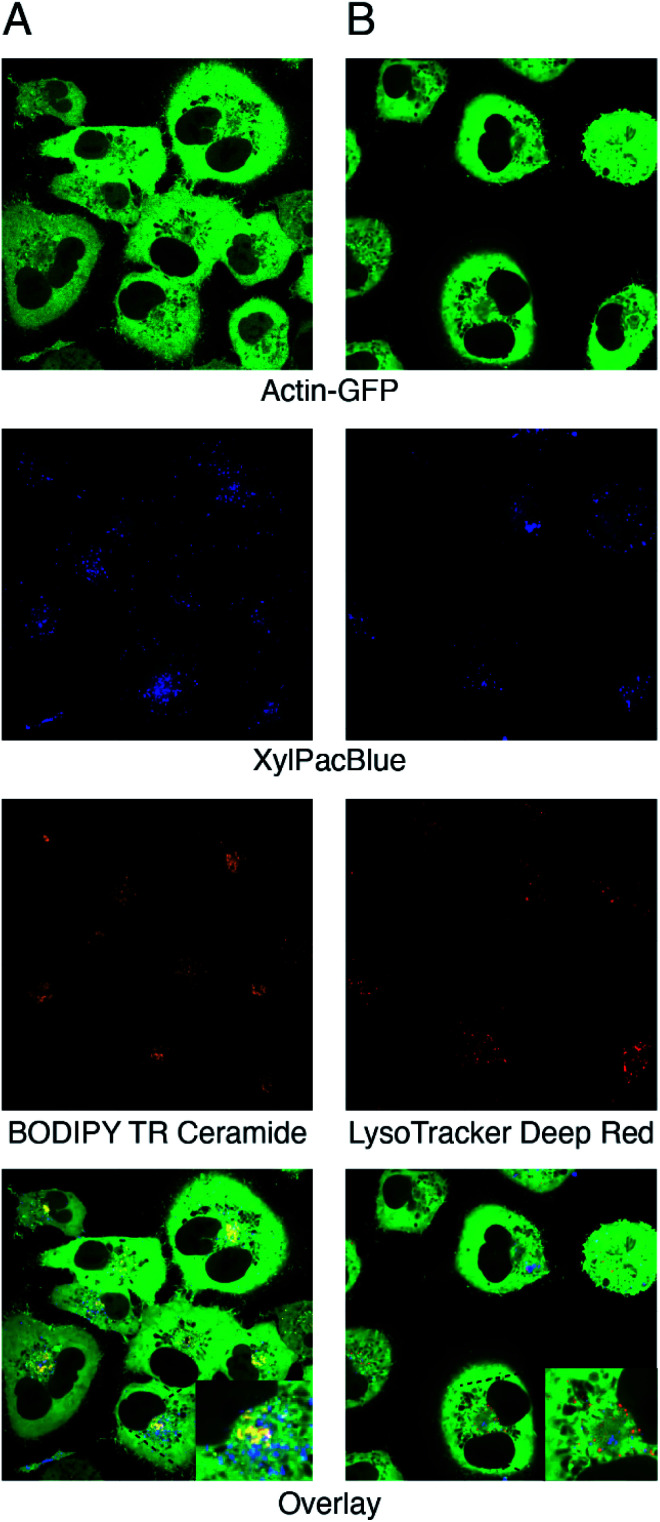
Confocal microscopy of GFP-tagged human A549 epithelial cells treated with XylPacBlue (5) and (A) BODIPY TR Ceramide which localizes to the Golgi apparatus or (B) LysoTracker Deep Red which targets lysosomes.

For visualization of uptake and processing of xylosides, the actin skeleton of A549 cells was tagged with green fluorescent protein (GFP). The GFP gene was inserted after the start codon of the human ACTB gene by CRISPR/Cas9 cleavage in the presence of a donor template, utilizing the natural homology-directed repair pathway. Confocal microscopy experiments after 24 hours of stimulation with 0.1 mM XylPacBlue (5) confirmed uptake and mostly perinuclear localization of the xyloside (Fig. 4). Using Golgi (Fig. 4A) and lysosome (Fig. 4B) trackers, we could confirm the lack of complete co-localization with both Golgi and lysosomes, suggesting that the XylPacBlue derivatives localize to the ER-Golgi intermediate compartment where linker region synthesis takes place.^44^ Our explanation for these observations is that Golgi entry and further polymerization might be inhibited by the low p*K*_a_ (3.7 for PacBlue hydroxyl) of these compounds.

## Conclusions

We conclude that compounds 5–9 all have good fluorescent ability and stability§§Compounds left in NMR-tubes dissolved in D_2_O for three weeks showed no apparent degradation or loss of fluorescence. and are galactosylated by β4GalT7. Furthermore, the compounds permeate A549 cell membranes and are galactosylated *in vivo* at least once and potentially twice. Interestingly, the biosynthesis is then terminated, and the compounds transported out of the cells. We hypothesize that the compounds are too charged (p*K*_a_ 3.7 for PacBlue hydroxyl) to enter the Golgi apparatus to any meaningful extent. The earlier reported compounds 3 and 4, that successfully enter cells and start the GAG synthesis do not have a charge on the fluorophores. We propose that the overall goal, *i.e.*, visualization and tracking of the GAG biosynthesis, may not be possible to reach by fluorescent probes but rather by utilizing compounds that are known to produce GAGs in cells, containing a linker that, after GAG-biosynthesis in the cells, could be modified to attach fluorescent groups.

## Experimental section

### Synthesis

All moisture- and air-sensitive reactions were carried out under an atmosphere of dry nitrogen using oven-dried glassware. All solvents were dried using MBRAUN SPS-800 Solvent purification system prior to use unless otherwise stated. Purchased reagents were used without further purification. Chromatographic separations were performed on Matrex silica gel (25–70 μm). Thin-layer chromatography was performed on precoated TLC glass plates with silica gel 60 F254 0.25 mm (Merck). Spots were visualized with UV light or by charring with an ethanolic anisaldehyde solution. Biotage Isolute phase separators were used for drying of combined organic layers. Preparative chromatography was performed on Biotage Isolera One flash purification system using Biotage SNAP KP-Sil silica cartridges or Agilent Technologies 1260 Infinity HPLC with Waters Symmetry C18 column, 5 μm, 19 × 100 mm was used for purification. Partition coefficient estimation was done using an Agilent Infinity 1260 with an Agilent SB-C18 column, 1.8 μm, 2.1 × 50 mm in a gradient of MeCN in H_2_O (with 0.1% formic acid in both solvents). Optical rotations were measured on a Bellingham and Stanley model ADP450 polarimeter and are reported as [*α*]^*T*^_D_ (*c* = g/100 mL), D indicate the sodium D line (589 nm) and *T* indicates the temperature. NMR spectra were recorded at ambient temperatures on a Bruker Avance II at 400 MHz (^1^H) and 100 MHz (^13^C) or a Bruker Ascend at 500 MHz (^1^H) and 125 MHz (^13^C) and assigned using 2D methods (COSY, HMQC). Chemical shifts are reported in ppm, with reference to residual solvent peaks (*δ*_H_ CHCl_3_ = 7.26 ppm, CD_3_OH = 3.31 ppm, C_6_D_5_H = 7.16 ppm) and solvent signals (*δ*_C_ CDCl_3_ = 77.0 ppm, CD_3_OD = 49.0 ppm, C_6_D_6_ = 128.06 ppm). Coupling constant values are given in Hz. Mass spectra were recorded on Waters XEVO G2 (Positive ESI).

### Procedure A (general glycosylation procedure)

Glycosyl acceptor (1 eq.) was dissolved in MeCN (dry) under a N_2_-atmosphere along with BF_3_·OEt_2_ (3 eq.). 1 (3 eq.) dissolved in a minimal amount of MeCN (dry) was added dropwise to the reaction vessel over 2 h. Water (100 mL) was added to the reaction vessel after 2 h and the solution extracted with DCM (3 × 50 mL). The DCM fraction was concentrated *in vacuo*. Purification by flash chromatography (SiO_2_, EtOAc : *n*-heptane 20 : 80 → 60 : 40), followed by recrystallisation from 2-propanol gave the product.

### Procedure B: (general hydrogenation procedure)

The acetylated carbohydrate intermediate (1 eq.) was dissolved in MeOH (dry). Pd/C (10 mol%, 100 mg g^−1^) was added, the flask evacuated, flushed with N_2_ (g), and the atmosphere was changed into H_2_ (g) (balloon). The reaction was stirred for 2 h after which the solution was filtered through Celite, and the solvent removed *in vacuo* to yield the product. No further purification was performed.

### Procedure C: (general amide coupling procedure)

The free amine intermediate (1 eq.) was dissolved in DCM (dry), and EDC (1.1 eq.), HOBt (1.1 eq., monohydrate), Pacific Blue™ carboxylic acid (1.1 eq.) and TEA (1.1 eq.) were added. The solution was stirred overnight, after which the solvent was removed *in vacuo*. The crude reaction mixture was purified by preparative HPLC and lyophilized to give the product.

### Procedure D (general deacetylation procedure)

The acetylated PacBlue-xyloside (1 eq.) was dissolved in MeOH and K_2_CO_3_ (0.1 eq.) was added. The solution was stirred for 1 h after which the solution was neutralized using Amberlite IR-120 exchange resin. The resin was filtered off, and the solvent removed *in vacuo* to yield the product. The crude was purified by preparative HPLC (H_2_O/MeCN 90 : 10 → 0 : 100) and lyophilized to give the final compound.

#### 2-((Benzyloxycarbonyl)amino)ethyl 2,3,4-tri-*O*-acetyl-β-d-xylopyranoside (11)

Following procedure A using benzyl (2-hydroxyethyl)carbamate (468 mg, 2.40 mmol) and 10 (636 mg, 2.00 mmol) yielded 11 as needle crystals (180 mg, 0.40 mmol, 20%, 100% β-anomer). mp 93.5–94.9 °C; [*α*]^25^_D_ −6.9 (*c* 1.0, CHCl_3_). ^1^H NMR (400 MHz, CDCl_3_) *δ* 7.39–7.31 (m, 5H, Ar–H), 5.18 (t, *J* = 8.8 Hz, 1H, H-3), 5.12 (s, 2H, CH_2_), 4.96 (td, *J* = 8.9, 5.2 Hz, 2H, H-4), 4.92 (dd, *J* = 8.9, 7.0 Hz, 1H, H-2), 4.48 (d, *J* = 7.0 Hz, 1H, H-1), 4.11 (dd, *J* = 11.8, 5.2 Hz, 1H, H-5_eq_), 3.86 (ddd, *J* = 9.9, 5.8, 4.0 Hz, 1H, –CH_2_–N), 3.65 (ddd, *J* = 10.2, 6.7, 3.8 Hz, 1H, –CH_2_–N), 3.48–3.39 (m, 2H, O–CH_2_–), 3.36 (dd, *J* = 11.8, 9.1 Hz, 1H, H-5_ax_), 2.07 (s, 3H, CH_3_), 2.05 (s, 3H, CH_3_), 2.03 (s, 3H, CH_3_). ^13^C NMR (101 MHz, CDCl_3_) *δ* 170.01, 169.79, 169.51, 156.33, 136.50, 128.51, 128.12, 128.10, 100.94, 71.46, 70.90, 68.82, 68.69, 66.74, 62.19, 40.81, 20.68, 20.65, 20.60. HRMS calcd for C_21_H_27_NO_10_ + H^+^ (M + H)^+^: 454.1711, found: 454.1713.

#### 2-Aminoethyl 2,3,4-tri-*O*-acetyl-β-d-xylopyranoside (12)

11 (180 mg, 0.40 mmol) was subjected to procedure B to yield 12 as an amorphous solid (128 mg, 0.40 mmol, 100%). [*α*]^25^_D_ −2.3 (*c* 0.4, MeOH). ^1^H NMR (400 MHz, MeOD) *δ* 5.25 (t, *J* = 9.2 Hz, 1H, H-3), 5.01–4.95 (m, 1H, H-2), 4.96 (dd, *J* = 9.3, 7.5 Hz, 1H, H-4), 4.71 (d, *J* = 7.5 Hz, 1H, H-1), 4.12 (dd, *J* = 11.7, 5.5 Hz, 1H, H-5_eq_), 4.00 (ddd, *J* = 10.7, 6.7, 4.0 Hz, 1H, –CH_2_–N), 3.88 (ddd, *J* = 11.5, 5.9, 4.0 Hz, 1H, –CH_2_–N), 3.51 (dd, *J* = 11.7, 9.8 Hz, 1H, H-5_ax_), 3.18–3.13 (m, 2H, O–CH_2_–), 2.06 (s, 3H, CH_3_), 2.04 (s, 3H, CH_3_), 2.02 (s, 3H, CH_3_). ^13^C NMR (101 MHz, MeOD) *δ* 170.16, 170.12, 170.07, 100.71, 72.03, 71.16, 68.88, 65.03, 62.04, 39.39, 19.22, 19.14, 19.08. HRMS calcd for C_13_H_21_NO_8_ + Na^+^ (M + Na)^+^: 342.1172, found: 342.1165.

#### 2-(6,8-Difluoro-7-hydroxycoumarin-3-carboxamido)ethyl 2,3,4-tri-*O*-acetyl-β-d-xylopyranoside (13)

12 (40 mg, 0.13 mmol) was subjected to procedure C to yield 13 as a yellow amorphous solid (25 mg, 0.046 mmol, 36%). [*α*]^25^_D_ −28.3 (*c* 0.4, MeCN). ^1^H NMR (400 MHz, CDCl_3_) *δ* 8.99 (t, *J* = 5.4 Hz, 1H, –NH–), 8.77 (d, *J* = 1.3 Hz, 1H, Ar–H), 7.23 (dd, *J* = 9.4, 2.0 Hz, 1H, Ar–H), 5.18 (t, *J* = 8.4 Hz, 1H, H-3), 5.01–4.95 (m, 2H, H-2, H-4), 4.58 (d, *J* = 6.7 Hz, 1H, H-1), 4.18 (dd, *J* = 11.9, 5.0 Hz, 1H, H-5_eq_), 3.98 (ddd, *J* = 10.2, 5.9, 3.9 Hz, 1H, –CH_2_–N), 3.78–3.61 (m, 3H, O–CH_2_–,–CH_2_–N), 3.42 (dd, *J* = 11.9, 8.6 Hz, 1H, H-5_ax_), 2.12 (s, 3H, CH_3_), 2.07 (s, 3H, CH_3_), 2.03 (s, 3H, CH_3_). ^13^C NMR (126 MHz, MeOD) *δ* 170.12, 170.00, 169.91, 147.92, 109.56, 100.35, 71.81, 70.97, 68.88, 67.33, 63.06, 39.20, 19.23, 19.10, 19.04. HRMS calcd for C_23_H_23_NO_12_F_2_ + Na^+^ (M + Na)^+^: 566.1085, found: 566.1086.

#### 2-(6,8-Difluoro-7-hydroxycoumarin-3-carboxamido)ethyl β-d-xylopyranoside (5)

13 (6.8 mg, 0.012 mmol) was subjected to procedure D to yield 5 as a yellow amorphous solid (5.0 mg, 0.012 mmol, 98%). [α]^25^_D_ 34.1 (*c* 0.26, MeOH). ^1^H NMR (500 MHz, MeOD) *δ* 9.19 (t, *J* = 5.5 Hz, 1H, –NH–), 8.75 (d, *J* = 1.4 Hz, 1H, Ar–H), 7.40 (dd, *J* = 10.2, 1.9 Hz, 1H, Ar–H), 4.27 (d, *J* = 7.5 Hz, 1H, H-1), 3.96 (ddd, *J* = 10.8, 6.7, 4.4 Hz, 1H, O–CH_2_–), 3.89 (dd, *J* = 11.5, 5.3 Hz, 1H, H-5_eq_), 3.80 (ddd, *J* = 10.5, 6.0, 4.4 Hz, 1H, O–CH_2_–), 3.71–3.61 (m, 2H, –CH_2_–N), 3.51 (ddd, *J* = 10.2, 8.8, 5.4 Hz, 1H, H-4), 3.25–3.19 (m, 2H, H-2, H-5_ax_). ^13^C NMR (126 MHz, MeOD) *δ* 162.90, 160.44, 150.48 (dd, *J* = 241.8, 4.3 Hz), 147.68, 144.59 (dd, *J* = 16.1, 12.8 Hz), 141.32 (d, *J* = 8.7 Hz), 139.69 (dd, *J* = 244.5, 6.1 Hz), 113.43, 109.46 (d, *J* = 21.2 Hz), 107.85 (d, *J* = 9.8 Hz), 103.85, 76.26, 73.40, 69.66, 67.92, 65.48, 39.55. HRMS calcd for C_17_H_17_NO_9_F_2_ + Na^+^ (M + Na)^+^: 440.0764, found: 440.0769.

#### 3-((Benzyloxycarbonyl)amino)propyl 2,3,4-tri-*O*-acetyl-β-d-xylopyranoside (14)

Following procedure A using benzyl (4-hydroxypropyl)carbamate (502 mg, 2.4 mmol) and 10 (636 mg, 2.0 mmol) to yield 14 as needle crystals (229 mg, 0.49 mmol 20%, 100% β-anomer). mp 96.1–97.0 °C; [*α*]^25^_D_ −19.2 (*c* 0.6, MeOH). ^1^H NMR (400 MHz, CDCl_3_) *δ* 7.38–7.28 (m, 5H, Ar), 5.16 (t, *J* = 8.6 Hz, 1H, H-3), 5.09 (s, 2H, –CH_2_–Ar), 5.00 (broad, 1H, –NH–), 4.96–4.87 (m, 2H, H-2, H-4) 4.46 (d, *J* = 6.8 Hz, 1H, H-1), 4.10 (dd, *J* = 11.8, 5.1 Hz, 1H, H-5_eq_), 3.88 (dt, *J* = 9.9, 5.9 Hz, 1H, O–CH_2_–), 3.54 (dt, *J* = 9.9, 5.9 Hz, 1H, O–CH_2_–), 3.35 (dd, *J* = 11.8, 8.8 Hz, 1H, H-5_ax_), 3.31–3.20 (m, 2H –CH_2_–N), 2.05 (s, 3H, Ac), 2.03 (s, 3H, Ac), 2.02 (s, 3H, Ac), 1.83–1.73 (m, 2H –CH_2_–). ^13^C NMR (101 MHz, CDCl_3_) *δ* 170.08, 169.85, 169.54, 156.41, 136.64, 128.52, 128.11, 128.08, 100.59, 71.35, 70.76, 68.82, 67.33, 66.59, 62.05, 38.46, 29.50, 20.75, 20.70, 20.69. HRMS calcd for C_22_H_29_NO_10_ + H^+^ (M + H)^+^: 468.1870, found: 468.1863.

#### 3-Aminopropyl 2,3,4-tri-*O*-acetyl-β-d-xylopyranoside (15)

14 (229 mg, 0.49 mmol) was subjected to procedure B to yield 15 as a clear oil (156 mg, 0.47 mmol, 96%). [*α*]^25^_D_ −5.7 (*c* 0.5, MeOH). ^1^H NMR (400 MHz, MeOD) *δ* 5.25 (t, *J* = 9.2 Hz, 1H, H-3), 4.98–4.93 (m, 2H, H-2, H-4), 4.62 (d, *J* = 7.4 Hz, 1H, H-1), 4.10 (dd, *J* = 11.7, 5.5 Hz, 1H, H-5_eq_), 3.96 (ddd, *J* = 10.1, 6.5, 5.2 Hz, 1H, O–CH_2_–), 3.74–3.67 (m, 1H, O–CH_2_–), 3.48 (dd, *J* = 11.7, 9.8 Hz, 1H, H-5_ax_), 3.04 (t, *J* = 7.1 Hz, 2H, –CH_2_–N), 2.07 (s, 3H, Ac), 2.04 (s, 3H, Ac), 2.03 (s, 3H, Ac), 1.98–1.90 (m, 2H, –CH_2_–). ^13^C NMR (101 MHz, MeOD) *δ* 170.15, 170.14, 168.46, 100.74, 71.80, 71.38, 69.01, 66.61, 61.93, 37.47, 27.13, 19.23, 19.15, 19.11. HRMS calcd for C_14_H_23_NO_8_ + H^+^ (M + H)^+^: 334.1503, found: 334.1502.

#### 3-(6,8-Difluoro-7-hydroxycoumarin-3-carboxamido)propyl 2,3,4-tri-*O*-acetyl-β-d-xylopyranoside (16)

15 (40 mg, 0.12 mmol) was subjected to procedure C to yield 16 as a yellow amorphous solid (28 mg, 0.048 mmol, 40%). [*α*]^25^_D_ −67.7 (*c* 0.53, MeCN). ^1^H NMR (400 MHz, MeOD) *δ* 9.01 (t, *J* = 5.7 Hz, 1H, –NH–), 8.74 (d, *J* = 1.4 Hz, 1H, Ar), 7.44 (dd, *J* = 10.2, 2.1 Hz, 1H, Ar), 5.20 (t, *J* = 9.0 Hz, 1H, H-3), 4.96–4.91 (m, 1H, H-4)*, 4.91 (t, *J* = 2.7 Hz, 1H, H-2)*, 4.61 (d, *J* = 7.3 Hz, 1H, H-1), 4.09 (dd, *J* = 11.7, 5.4 Hz, 1H, H-5_eq_), 3.91 (dt, *J* = 10.0, 5.9 Hz, 1H, O–CH_2_–), 3.65 (dt, *J* = 10.0, 5.9 Hz, 1H, O–CH_2_–), 3.53–3.47 (m, 2H, –CH_2_–N), 3.46 (dd, *J* = 11.7, 9.5 Hz, 1H, H-5_ax_), 2.03 (s, 3H), 2.02 (s, 3H), 2.00 (s, 3H), 1.87 (p, *J* = 6.2 Hz, 2H, –CH_2_–). ^13^C NMR (101 MHz, MeOD) *δ* 170.25, 170.12, 169.87, 162.45, 160.31, 147.42, 115.33, 109.87, 109.84, 109.81, 109.63, 109.35, 100.76, 71.99, 71.22, 69.06, 66.92, 61.77, 36.62, 28.83, 19.24, 19.19, 19.14. HRMS calcd for C_24_H_25_NO_12_F_2_ + Na^+^ (M + Na)^+^: 580.1229, found: 580.1243. *Overlapping with solvent peak, assigned with COSY experiment.

#### 3-(6,8-Difluoro-7-hydroxycoumarin-3-carboxamido)propyl β-d-xylopyranoside (6)

16 (6.1 mg, 0.011 mmol) was subjected to procedure D to yield 6 as a yellow amorphous solid (4.8 mg, 0.011 mmol, 96%) [*α*]^25^_D_ −12.0 (*c* 0.81, MeOH). ^1^H NMR (400 MHz, MeOD) *δ* 9.12 (t, *J* = 5.6 Hz, 1H, –NH–), 8.75 (d, *J* = 1.4 Hz, 1H, Ar), 7.42 (dd, *J* = 10.2, 2.0 Hz, 1H, Ar), 4.23 (d, *J* = 7.4 Hz, 1H, H-1), 3.94 (ddd, *J* = 10.0, 6.8, 5.4 Hz, 1H, O–CH_2_–), 3.89 (dd, *J* = 11.4, 5.4 Hz, 1H, H-5_eq_), 3.73–3.66 (m, 1H, O–CH_2_–), 3.57 (dt, *J* = 10.8, 5.5 Hz, 2H, –CH_2_–N), 3.51 (ddd, *J* = 10.3, 7.8, 4.5 Hz, 1H, H-5_ax_), 3.36–3.19 (m, 3H, H-2, H-3, H-4)*, 1.97–1.88 (m, 2H, –CH_2_–). ^13^C NMR (101 MHz, MeOD) *δ* 162.59, 160.45, 147.59, 114.37, 109.75, 108.63, 103.85, 76.48, 73.47, 69.82, 67.16, 65.62, 36.88, 29.00. HRMS calcd for C_18_H_19_NO_9_F_2_ + H^+^ (M + H)^+^: 432.1106, found: 432.1097. *Overlapping with solvent peak, assigned with COSY experiment.

#### 4-((Benzyloxycarbonyl)amino)butyl 2,3,4-tri-*O*-acetyl-β-d-xylopyranoside (17)

Following procedure A using benzyl (4-hydroxybutyl)carbamate (536 mg, 2.4 mmol) and 10 (636 mg, 2.0 mmol) to yield 17 as needle crystals (134 mg, 0.28 mmol, 14%, 100% β-anomer). mp 96.2–98.5 °C; [*α*]^25^_D_ −16.4 (*c* 0.7, MeOH). ^1^H NMR (400 MHz, CDCl_3_) *δ* 7.39–7.31 (m, 5H, Ar), 5.18 (t, *J* = 8.6 Hz, 1H, H-3), 5.11 (s, 2H, –CH_2_–), 4.96 (td, *J* = 8.8, 5.1 Hz, 1H, H-4), 4.92 (dd, *J* = 8.7, 6.9 Hz, 1H, H-2), 4.85 (broad, s, 1H, –NH–), 4.47 (d, *J* = 6.8 Hz, 1H, H-1), 4.12 (dd, *J* = 11.8, 5.1 Hz, 1H, H-5_eq_), 3.85 (dt, *J* = 9.9, 5.9 Hz, 1H, O–CH_2_), 3.51 (dt, *J* = 9.6, 5.7 Hz, 1H, O–CH_2_), 3.36 (dd, *J* = 11.8, 8.9 Hz, 1H, H-5_ax_), 3.23 (q, *J* = 6.3 Hz, 2H, –CH_2_–N), 2.07 (s, 3H, Ac), 2.06 (s, 3H, Ac), 2.05 (s, 3H, Ac), 1.65–1.56 (m, 4H, –CH_2_–CH_2_–). ^13^C NMR (101 MHz, CDCl_3_) *δ* 170.06, 169.81, 169.43, 156.40, 136.66, 128.51, 128.07, 100.70, 71.51, 70.91, 69.06, 68.92, 66.60, 62.07, 40.63, 26.61, 26.57, 20.71, 20.68, 20.67. HRMS calcd for C_23_H_31_NO_10_ + H^+^ (M + H)^+^: 482.2024, found: 482.2026.

#### 3-Aminobutyl 2,3,4-tri-*O*-acetyl-β-d-xylopyranoside (18)

17 (134 mg, 0.28 mmol) was subjected to procedure B to yield 18 as a clear sticky solid (91 mg, 0.027 mmol, 94%) [*α*]^25^_D_ −4.2 (*c* 0.7, MeOH). ^1^H NMR (400 MHz, MeOD) *δ* 5.23 (t, *J* = 9.2 Hz, 1H, H-3), 4.96–4.92 (m, 1H, H-4)*, 4.86 (dd, *J* = 9.3, 7.4 Hz, 1H, H-2), 4.60 (d, *J* = 7.4 Hz, 1H, H-1), 4.08 (dd, *J* = 11.7, 5.4 Hz, 1H, H-5_eq_), 3.88 (dt, *J* = 9.8, 5.7 Hz, 1H, O–CH_2_–), 3.61 (dt, *J* = 9.8, 5.7 Hz, 1H O–CH_2_–), 3.47 (dd, *J* = 11.7, 9.8 Hz, 1H, H-5_ax_), 2.95 (t, *J* = 6.8 Hz, 2H, CH_2_–N), 2.06 (s, 3H, Ac), 2.03 (s, 3H, Ac), 2.02 (s, 3H, Ac), 1.78–1.65 (m, 4H, –CH_2_–CH_2_–). ^13^C NMR (101 MHz, MeOD) *δ* 170.16, 170.13, 169.96, 100.77, 71.97, 71.39, 69.06, 68.50, 61.87, 39.16, 26.10, 24.32, 19.22, 19.15, 19.10. HRMS calcd for C_15_H_25_NO_8_ + H^+^ (M + H)^+^: 348.1659, found: 348.1658. *Overlapping with solvent peak, assigned with COSY experiment.

#### 4-(6,8-Difluoro-7-hydroxycoumarin-3-carboxamido)butyl 2,3,4-tri-*O*-acetyl-β-d-xylopyranoside (19)

18 (41.4 mg, 0.121 mmol) was subjected to procedure C to yield 19 as a yellow amorphous solid (41.5 mg, 0.0698 mmol, 58%) [*α*]^25^_D_ 35.2 (*c* 0.39, MeCN). ^1^H NMR (400 MHz, MeOD) *δ* 8.98 (t, *J* = 5.6 Hz, 1H, –NH–), 8.77 (d, *J* = 1.5 Hz, 1H, Ar), 7.48 (dd, *J* = 10.2, 2.1 Hz, 1H, Ar), 5.20 (t, *J* = 9.0 Hz, 1H, H-3), 4.93 (dd, *J* = 9.5, 5.4 Hz, 1H, H-4)*, 4.86 (dd, *J* = 9.1, 7.3 Hz, 1H, H-2)*, 4.60 (d, *J* = 7.3 Hz, 1H, H-1), 4.07 (dd, *J* = 11.7, 5.4 Hz, 1H, H-5_eq_), 3.92–3.85 (m, 1H, O–CH_2_–), 3.62–3.55 (m, 1H, O–CH_2_–), 3.46 (m, 2H, CH_2_–N), 3.46 (dd, *J* = 11.7, 9.5 Hz, 1H, H-5_ax_), 2.04 (s, 3H, Ac), 2.03 (s, 3H, Ac), 2.01 (s, 3H, Ac), 1.72–1.66 (m, 4H, –CH_2_–CH_2_–). ^13^C NMR (101 MHz, MeOD) *δ* 170.22, 170.11, 169.85, 162.34, 147.44, 115.63, 109.93, 100.66, 71.94, 71.23, 69.05, 68.77, 61.70, 38.94, 26.50, 25.71, 19.25, 19.18, 19.13. HRMS calcd for C_25_H_27_NO_12_F_2_ + H^+^ (M + H)+: 594.1396, found: 594.1399. *Partially overlapping with solvent peak, assigned with COSY experiment.

#### 4-(6,8-Difluoro-7-hydroxycoumarin-3-carboxamido)butyl β-d-xylopyranoside (7)

19 (6.5 mg, 0.011 mmol) was subjected to procedure D to yield 7 as a yellow amorphous solid (4.8 mg, 0.011 mmol, 96%). [*α*]^25^_D_ +63.8 (*c* 0.70, MeOH). ^1^H NMR (500 MHz, MeOD) *δ* 9.00 (t, *J* = 5.7 Hz, 1H, –NH–), 8.76 (d, *J* = 1.5 Hz, 1H, Ar), 7.46 (dd, *J* = 10.1, 2.0 Hz, 1H, Ar), 4.22 (d, *J* = 7.6 Hz, 1H, H-1), 3.89 (dt, *J* = 9.8, 6.0 Hz, 1H, O–CH_2_–), 3.86 (dd, *J* = 11.5, 5.4 Hz, 1H, H-5_eq_), 3.61 (dt, *J* = 9.8, 5.9 Hz, 1H, O–CH_2_–), 3.52–3.45 (m, 3H, H-4, –CH_2_–N), 3.32–3.29 (m, 1H, H-3), 3.24–3.16 (m, 2H, H-2, H-5_ax_), 1.80–1.69 (m, 4H, –CH_2_–CH_2_–). ^13^C NMR (126 MHz, MeOD) *δ* 162.38, 160.29, 147.41, 109.57, 103.68, 76.40, 73.47, 69.75, 68.84, 65.47, 38.97, 26.55, 25.65. HRMS calcd for C_19_H_21_NO_9_F_2_ + Na^+^ (M + Na)^+^: 468.1078, found: 468.1082.

#### ((2*R*)-(Benzyloxycarbonyl)amino)propyl 2,3,4-tri-*O*-acetyl-β-d-xylopyranoside (20)

Following procedure A using benzyl (*R*)-(1-hydroxypropan-2-yl)carbamate (536 mg, 2.4 mmol) and 10 (636 mg, 2.0 mmol) to yield 20 as needle crystals (236 mg, 0.50 mmol, 21%, 100% β-anomer). mp 86.3–89.3 °C; [*α*]^25^_D_ −18.6 (*c* 0.8, MeOH). ^1^H NMR (400 MHz, CDCl_3_) *δ* 7.39–7.32 (m, 5H, Ar), 5.18 (t, *J* = 8.5 Hz, 1H, H-3), 5.11 (s, 2H, –CH_2_–), 4.96 (dd, *J* = 8.6, 5.0 Hz, 1H, H-4), 4.93 (dd, *J* = 8.7, 6.7 Hz, 1H, H-2), 4.48 (d, *J* = 6.7 Hz, 1H, H-1), 4.12 (dd, *J* = 11.8, 5.1 Hz, 1H, H-5_eq_), 3.98–3.91 (m, 1H, O–CH_2_–), 3.77 (dd, *J* = 9.6, 3.9 Hz, 1H, CH), 3.57–3.52 (m, 1H, O–CH_2_–), 3.37 (dd, *J* = 11.6, 9.0 Hz, 1H, H-5_ax_), 2.07 (s, 3H, Ac), 2.05 (s, 3H, Ac), 2.01 (s, 3H, Ac), 1.21 (d, *J* = 6.8 Hz, 3H, CH_3_). ^13^C NMR (101 MHz, CDCl_3_) *δ* 170.01, 169.85, 169.59, 155.65, 136.47, 128.53, 128.15, 100.78, 72.20, 71.18, 70.68, 68.75, 66.65, 62.00, 46.49, 20.75, 20.71, 20.61, 17.80. C_22_H_29_NO_10_ + H^+^ (M + H)^+^: 468.1864, found: 468.1870.

#### (2*R*)-2-Aminopropyl 2,3,4-tri-*O*-acetyl-β-d-xylopyranoside (21)

20 (236 mg, 0.505 mmol) was subjected to procedure B to yield 21 as a clear sticky solid (103 mg, 0.310 mmol, 62%). [*α*]^25^_D_ −6.3 (*c* 0.9, MeOH). ^1^H NMR (400 MHz, MeOD) *δ* 8.45 (s, 1H, NH_2_, exchangeable), 5.25 (t, *J* = 9.1 Hz, 1H, H-3), 5.01–4.94 (m, 2H, H-2, H-4)*, 4.71 (d, *J* = 7.4 Hz, 1H, H-1), 4.12 (dd, *J* = 11.7, 5.4 Hz, 1H, H-5_eq_), 3.79 (d, *J* = 5.9 Hz, 2H, O–CH_2_–), 3.55–3.44 (m, 2H, H-5_ax_, CH), 2.06 (s, 3H, Ac), 2.04 (s, 3H, Ac), 2.03 (s, 3H, Ac), 1.29 (d, *J* = 6.8 Hz, 3H, CH_3_). ^13^C NMR (101 MHz, MeOD) *δ* 170.15, 170.13, 170.07, 167.43, 100.21, 71.91, 71.05, 69.43, 68.84, 61.95, 19.25, 19.17, 19.12, 13.77. C_14_H_23_NO_8_ + Na^+^ (M + Na)^+^: 334.1500, found: 334.1502. *Partially overlapping with solvent peak, assigned by COSY experiment.

#### ((2*R*)-2-(6,8-Difluoro-7-hydroxycoumarin-3-carboxamido))propyl 2,3,4-tri-*O*-acetyl-β-d-xylopyranoside (22)

21 (40 mg, 0.12 mmol) was subjected to procedure C to yield 22 as a bright yellow solid (38 mg, 0.066 mmol, 55%). [*α*]^25^_D_ −58.4 (*c* 0.40, MeCN). ^1^H NMR (400 MHz, MeOD) *δ* 9.02 (d, *J* = 7.9 Hz, 1H, –NH–), 8.78 (d, *J* = 1.4 Hz, 1H, Ar), 7.47 (dd, *J* = 10.2, 2.0 Hz, 1H, Ar), 5.12 (t, *J* = 8.8 Hz, 1H, H-3), 5.00–4.92 (m, 2H, H-2, H-4)*, 4.66 (d, *J* = 7.1 Hz, 1H, H-1), 4.35–4.26 (m, 1H, CH), 4.11 (dd, *J* = 11.7, 5.3 Hz, 1H, H-5_eq_), 3.91 (dd, *J* = 9.9, 4.4 Hz, 1H, O–CH_2_–), 3.63 (dd, *J* = 9.9, 4.2 Hz, 1H, O–CH_2_–), 3.49 (dd, *J* = 11.8, 9.3 Hz, 1H, H-5_ax_), 2.08 (s, 3H, Ac), 2.03 (s, 3H, Ac), 2.02 (s, 3H, Ac), 1.28 (d, *J* = 6.8 Hz, 3H, CH_3_). ^13^C NMR (101 MHz, MeOD) *δ* 170.21, 170.14, 170.06, 161.75, 160.32, 147.71, 109.71, 100.40, 71.78, 71.19, 70.99, 68.97, 61.64, 45.33, 19.37, 19.18, 19.13, 16.10. HRMS calcd for C_24_H_25_NO_12_F_2_ + Na^+^ (M + Na)^+^: 580.1241, found: 580.1243. *Overlapping with solvent peak, assigned by COSY experiment.

#### ((2*R*)-2-(6,8-Difluoro-7-hydroxycoumarin-3-carboxamido))propyl β-d-xylopyranoside (8)

22 (10.1 mg, 0.018 mmol) was subjected to procedure D to yield 8 (5.7 mg, 0.013 mmol, 73%) as a bright yellow solid. [*α*]^25^_D_ −114 (*c* 0.1, MeOH). ^1^H NMR (400 MHz, MeOD) *δ* 8.98 (d, *J* = 8.1 Hz, 1H, –NH–), 8.76 (d, *J* = 1.4 Hz, 1H, Ar), 7.46 (dd, *J* = 10.2, 2.0 Hz, 1H, Ar), 4.39–4.29 (m, 1H, –CH–), 4.26 (d, *J* = 7.5 Hz, 1H, H-1), 3.91–3.85 (m, 2H, O–CH_2_–, H-5_eq_), 3.67 (dd, *J* = 10.2, 4.7 Hz, 1H, O–CH_2_–), 3.53–3.45 (m, 1H, H-5_ax_), 3.33 (m, 1H, H-3)* 3.27–3.16 (m, 2H, H-2,H-4), 1.31 (d, *J* = 6.8 Hz, 3H, –CH_3_). ^13^C NMR (101 MHz, MeOD) *δ* 162.06, 160.32, 147.55, 115.24, 109.88, 103.95, 76.31, 73.45, 72.13, 69.75, 65.55, 45.67, 16.11. HRMS calcd for C_18_H_19_NO_9_F_2_ + Na^+^ (M + Na)^+^: 454.0926, found: 454.0921. *Overlapping with solvent peak, assigned by COSY experiment.

#### 2-((*tert*-Butyloxycarbonyl)amino)ethyl 2,3,4-tri-*O*-acetyl-1,5-dithio-β-d-xylopyranoside (24)

ZnCl_2_ (55.1 mg, 0.67 mmol), ZnO (90.0 mg, 0.67) and 2-(BOC-amino)ethanethiol (361 mg, 2.0 mmol) were dissolved in a 1 : 1 mixture of MeCN and toluene (20 mL), together with molecular sieves (3 Å). The mixture was heated to 50 °C for 20 minutes, after which 23 (180 mg, 0.51 mmol) was added. The reaction was kept at 50 °C for 2 h. The mixture was then diluted with DCM (40 mL), washed with NaHCO_3_ (2 × 30 mL, sat. aq.) and brine (2 × 30 mL). The organic phase was dried and concentrated *in vacuo*. Purification by flash chromatography (39 : 59 : 2 EtOAc/*n*-heptanes/TEA) yielded 24 (92 mg, 40%, 2 : 1 β : α) as an amorphous solid. ^1^H NMR (400 MHz, CDCl_3_) *δ* 5.37 (t, *J* = 9.8 Hz, 1H, H-4), 5.14–5.07 (m, 1H, H-2), 5.06–5.03 (m, 1H, H-3), 3.77 (d, *J* = 10.3 Hz, 1H, H-1), 3.37–3.29 (m, 2H, –CH_2_–N), 2.86 (t, *J* = 6.5 Hz, 2H, S–CH_2_–), 2.76–2.71 (m, 2H, H-5), 2.02 (m, 9H, Ac), 1.44 (s, 9H, *t*-Bu). ^13^C NMR (101 MHz, CDCl_3_) *δ* 169.73, 169.71, 169.64, 73.93, 73.00, 72.37, 70.22, 48.49, 40.07, 31.45, 31.37, 28.40, 20.62, 20.60, 20.55. HRMS calcd for C_18_H_29_NO_8_S_2_ + Na^+^ (M + Na)^+^: 474.1231, found: 474.1232.

#### 2-((*tert*-Butyloxycarbonyl)amino)ethyl 2,3,4-tri-*O*-acetyl-1,5-dithio-α-d-xylopyranoside

^1^H NMR (400 MHz, CDCl_3_) *δ* 5.37 (t, *J* = 9.8 Hz, 1H, H-3), 5.22 (dd, *J* = 10.2, 4.5 Hz, 1H, H-2), 5.02–4.98 (m, 1H, H-4), 4.45 (dd, *J* = 4.5, 1.5 Hz, 1H, H-1), 3.37–3.28 (m, 2H, –CH_2_–N)*, 3.12 (dd, *J* = 13.3, 11.3 Hz, 1H, H-5_eq_), 2.75–2.71 (m, 2H, S–CH_2_–), 2.68–2.62 (m, 1H, H-5_ax_), 2.07 (s, 3H, Ac), 2.03 (s, 3H, Ac), 2.02 (s, 3H, Ac), 1.44 (s, 9H, *t*-Bu)*. ^13^C NMR (101 MHz, CDCl_3_) *δ* 169.89, 74.64, 73.39, 72.99, 70.22, 49.76, 40.06, 25.67′, 25.09′, 28.37, 20.83, 20.79. HRMS calcd for C_18_H_29_NO_8_S_2_ + Na^+^ (M + Na)^+^: 474.1231, found: 474.1232. *Overlapping with corresponding peak for β-anomer. Assigned with HSQC experiment.

#### 2-((*tert*-Butyloxycarbonyl)amino)ethyl 1,5-dithio-β-d-xylopyranoside (25)

24 (88.0 mg, 0.20 mmol) was dissolved in MeOH (10 mL). LiOH (18.7 mg, 0.78 mmol) was added, and the mixture was stirred for 40 minutes. The mixture was neutralized with Amberlite IR120 (H^+^) which was then filtered of. The mixture was concentrated *in vacuo* and purified by flash chromatography (9 : 1 DCM : EtOH with 2% TEA) to yield 25 (62.5 mg, 98%, 2 : 1 β : α) as an amorphous solid. ^1^H NMR (400 MHz, CDCl_3_) *δ* 3.82 (ddd, *J* = 10.2, 9.4, 5.1 Hz, 1H, H-4), 3.65 (d, *J* = 10.2 Hz, 1H, H-1), 3.52–3.50 (m, 1H, H-2), 3.38 (t, *J* = 6.1 Hz, 2H, S–CH_2_–), 3.28 (t, *J* = 8.9 Hz, 1H, H-3), 3.12 (qd, *J* = 7.3, 4.9 Hz, 2H, –CH_2_–N), 2.73–2.67 (m, 2H, H-5), 1.45 (s, 9H, *t*-Bu). ^13^C NMR (101 MHz, CDCl_3_) *δ* 156.65, 79.17, 75.50, 73.46, 72.57, 50.79, 45.92, 33.51, 28.44, 28.41. HRMS calcd for C_12_H_23_NO_5_S_2_ + H^+^ (M + H)^+^: 326.1096, found: 326.1090.

#### 2-((*tert*-Butyloxycarbonyl)amino)ethyl 1,5-dithio-α-d-xylopyranoside

^1^H NMR (400 MHz, CDCl_3_) *δ* 4.16 (d, *J* = 3.3 Hz, 1H, H-1), 4.04 (dd, *J* = 9.4, 4.3 Hz, 1H, H-2), 3.78–3.76 (m, 1H, H-4), 3.55–3.49 (m, 1H, H-3), 3.36–3.32 (m, 2H, S–CH_2_–)*, 3.07–2.96 (m, 1H, H-5_eq_), 2.89–2.82 (m, 2H, –CH_2_–N), 2.63–2.58 (m, 1H, H-5_ax_), 1.44 (s, 9H, *t*-Bu)*. ^13^C NMR (101 MHz, CDCl_3_) *δ* 75.05, 73.46, 53.32, 34.46, 31.99, 28.44. HRMS calcd for C_12_H_23_NO_5_S_2_ + H^+^ (M + H)^+^: 326.1096, found: 326.1090. *Overlapping with corresponding peak for β-anomer.

#### 2-Aminoethyl 1,5-dithio-β-d-xylopyranoside (26)

25 (62.5 mg, 0.191 mmol) was dissolved in EtOH (5 mL). HCl (1 M, 5 mL) was added, and the mixture was stirred for 1 h, after which the solvent was removed *in vacuo*. The residue was purified by preparatory HPLC (H_2_O : MeCN 90 : 10 → 0 : 100) and lyophilized to yield β-26 (22.5 mg, 0.10 mmol, 52%) as an amorphous solid. [*α*]^25^_D_ +51.0 (*c* 0.3, D_2_O). ^1^H NMR (400 MHz, D_2_O) *δ* 3.86 (d, *J* = 10.3 Hz, 1H, H-1), 3.74 (td, *J* = 9.6, 5.8 Hz, 1H, H-4), 3.47 (t, *J* = 9.8 Hz, 1H, H-2), 3.28 (t, *J* = 6.9 Hz, 2H, H-2), 3.24 (t, *J* = 9.5 Hz, 2H, S–CH_2_–), 3.09 (dt, *J* = 14.3, 7.0, 1H, H-5_eq_), 2.99 (dt, *J* = 14.3, 7.0 Hz, 1H, H-5_ax_), 2.71 (dd, *J* = 7.8, 3.5 Hz, 2H, –CH_2_–N). ^13^C NMR (101 MHz, D_2_O) *δ* 78.12, 75.50, 72.55, 49.36, 38.95, 33.00, 28.10. HRMS calcd for C_7_H_15_NO_3_S_2_ + H^+^ (M + H)^+^: 226.0572, found: 226.0565.

#### 2-(6,8-Difluoro-7-hydroxycoumarin-3-carboxamido)ethyl 1,5-dithio-β-d-xylopyranoside (9)

26 (5.1 mg, 0.023 mmol) was dissolved in DMF (3 mL) along with Pacific Blue™ succinimidyl ester (15.8 mg, 0.047 mmol). DIPEA (4.0 μL, 0.093 mmol) was added and the reaction was stirred for 4 h at r.t. The solvent was removed *in vacuo*. The residue was purified by preparatory HPLC (H_2_O : MeCN 90 : 10 → 0 : 100) and lyophilized to yield 9 as a yellow amorphous solid (4.5 mg, 0.010 mmol, 46%). [*α*]^25^_D_ −146 (*c* 0.72, MeOH). ^1^H NMR (500 MHz, MeOD) *δ* 9.23 (t, *J* = 5.8 Hz, 1H, –NH–), 8.71 (d, *J* = 1.5 Hz, 1H, Ar), 7.31 (dd, *J* = 10.4, 1.8 Hz, 1H, Ar), 3.79 (d, *J* = 10.2 Hz, 1H, H-1), 3.69 (td, *J* = 6.8, 3.2 Hz, 2H, S–CH_2_–), 3.64 (ddd, *J* = 10.8, 9.1, 4.5 Hz, 1H, H-4), 3.38 (dd, *J* = 10.2, 8.7 Hz, 1H, H-2), 3.15 (t, *J* = 8.9 Hz, 1H, H-3), 3.02–2.91 (m, 2H, –CH_2_–N), 2.68 (dd, *J* = 13.4, 10.9 Hz, 1H, H-5_eq_), 2.58 (dd, *J* = 13.4, 4.5 Hz, 1H, H-5_ax_). ^13^C NMR (126 MHz, MeOD) *δ* 163.42, 161.10, 151.75 (dd, *J* = 241.2, 6.5 Hz), 147.98, 141.84 (d, *J* = 8.9 Hz), 140.41 (dd, *J* = 241.6, 7.9 Hz), 110.79, 109.12 (dd, *J* = 21.4, 2.0 Hz), 105.89 (d, *J* = 10.8 Hz), 78.95, 76.23, 72.97, 50.55, 39.42, 33.41, 30.46. HRMS calculated for C_17_H_17_NO_7_S_2_F_2_ + H^+^ (M + H)^+^, 450.0493; found: 450.0486.

### β4GalT7 assay

The β4GalT7 enzymatic assay was performed as previously described.^41^ Briefly, β4GalT7 (50 ng) was mixed in 96-well polypropylene plates with UDP-Gal (1 mM final concentration) and various concentrations of xylosides in a final volume of 50 μL MES buffer (20 mM, pH 6.2) supplemented with MnCl_2_ (10 mM). Incubation was performed at 37 °C for 30 min and the reaction was stopped by cooling at 4 °C and addition of HPLC eluent (70% NH_4_OAc (60 mM, pH 5.6) – 30% CH_3_CN (v/v)) before HPLC analysis.

### GFP tagging of the ACTB gene in human A549 epithelial cells

To N-terminally tag the human actin protein (cytoplasmic 1, encoded by ACTB) of A549 cells with GFP, we used the Invitrogen TrueTag DNA donor kit (Thermo Fisher Scientific) which utilizes the natural homology directed repair pathway. CRISPR/Cas9 cleavage was performed in the presence of a donor template containing puromycin resistance and Emerald GFP genes (N-Puro-EmGFP), with ACTB-matching homology arms on each side of the inserted genes. The following primers, with 2× phosphorothioate modifications at the 5′ ends, were used to PCR amplify the donor template (non-bolded bases homologous to the ACTB gene and bolded homologous to N-Puro-EmGFP):
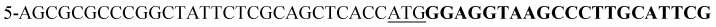


5′-TTGTCGACGACGAGCGCGGCGATATCATCATC**ACCGCTTCCACTACCTGAACC**

The purified PCR product was co-transfected with the TrueCut Cas9 v2 protein and a TrueGuide synthetic guide RNA (GCGGCGAUAUCAUCAUCCA, TGG PAM site) into A549 cells (ATCC CCL-185) using Lipofectamine CRISPRMAX Cas9 transfection reagent.

Modified cells were selected by FACS and verified using the following junction primers:

ACTB-F primer: 5′-GACGCCTCCGACCAGTGTTTGCC.

N-Puro-EmGFP-R: 5′-GGGTAATCGGCGAAGGCAGCGG.

### Xyloside stimulation of A549 cells

Actin-EmGFP-modified A549 cells were cultured to approx. 70% confluence in DMEM/F-12/GlutaMAX (Thermo Fisher Scientific) supplemented with 10% FBS (Thermo Fisher Scientific), 100 units per mL penicillin and 100 μg mL^−1^ streptomycin (Sigma-Aldrich). For xyloside treatment, the growth medium was aspirated and xylosides (100 μM from 50 mM stock solution in DMSO) in OptiPRO SFM medium (Thermo Fisher Scientific) was added to the cells. After 24 h of treatment, medium samples were either analyzed directly by fluorescence detection size-exclusion chromatography (FSEC) on an AdvanceBio SEC column (Agilent) or purified on an anion exchange DE52 diethylaminoethyl cellulose resin (Sigma-Aldrich). Purified samples were subsequently analyzed by FSEC or reversed-phase chromatography on an ACE C8, 3 μm 4.6 × 100 mm (ACE).

### Confocal microscopy of A549 cells

Localization experiments were performed on a Nikon Confocal A1RHD microscope with cells seeded on Lab-Tek 8-well (0.8 cm^2^) borosilicate (0.17 mm) slides (Thermo Fisher Scientific). For staining of lysosomes, LysoTracker Deep Red (Thermo Fisher Scientific) was used at a final concentration of 50 nM. Staining of the Golgi apparatus was performed using BODIPY TR Ceramide complexed to BSA (Thermo Fisher Scientific) at a final concentration of 5 μM. Compound 5 was used at a final concentration of 100 μM. Cells were left together with the substances at 37 °C for 100 min, after which the cell layer was washed twice with Dulbecco's Phosphate Buffered Saline (Sigma-Aldrich) and then covered with OptiPRO SFM medium before imaging.

## Author contributions

Roberto Mastio: investigation, formal analysis, visualization, writing – original draft, writing – review & editing. Daniel Willén: conceptualization, supervision, investigation, visualization, writing – original draft, writing – review & editing. Zackarias Söderlund: investigation, methodology, writing – review & editing. Gunilla Westergren-Thorsson: supervision, writing – review & editing, Sophie Manner: supervision, writing – review & editing. Emil Tykesson: investigation, supervision, visualization, writing – original draft, writing – review & editing. Ulf Ellervik: conceptualization, funding acquisition, supervision, visualization, writing – original draft, writing – review & editing.

## Conflicts of interest

There are no conflicts to declare.

## Supplementary Material

RA-011-D1RA06320K-s001
