# Supplementary material for: Fluorescently labeled xylosides offer insight into the biosynthetic pathways of glycosaminoglycans
Source: RSC Adv. 2021 Nov 29;11(60):38283–92. doi: 10.1039/d1ra06320k (PMC9044174; doi:10.1039/d1ra06320k)
Supplement: RA-011-D1RA06320K-s001 [file RA-011-D1RA06320K-s001.pdf]

Electronic Supplementary Material (ESI) for RSC Advances.
This journal is © The Royal Society of Chemistry 2021

Supporting Information

Fluorescently labeled xylosides offer insight into the biosynthetic pathways of glycosaminoglycans.

Roberto Mastio[1], Daniel Willén[1], Zackarias Söderlund[2], Gunilla Westergren-Thorsson[2], Sophie Manner[1], Emil Tykesson[2], Ulf Ellervik[1,2*]

[1]Centre for Analysis and Synthesis, Centre for Chemistry and Chemical Engineering, Lund University, P.O. Box 124, SE-221 00 Lund, Sweden.

[2]Department of Experimental Medical Science, Lund University, P.O. Box 117, SE-221 00 Lund, Sweden

* Corresponding author. *E-mail address:* ulf.ellervik@chem.lu.se.

1. Chromatographic analysis S2-S3
2. ^{1}H-NMR and ^{13}C-NMR spectra S4-S23

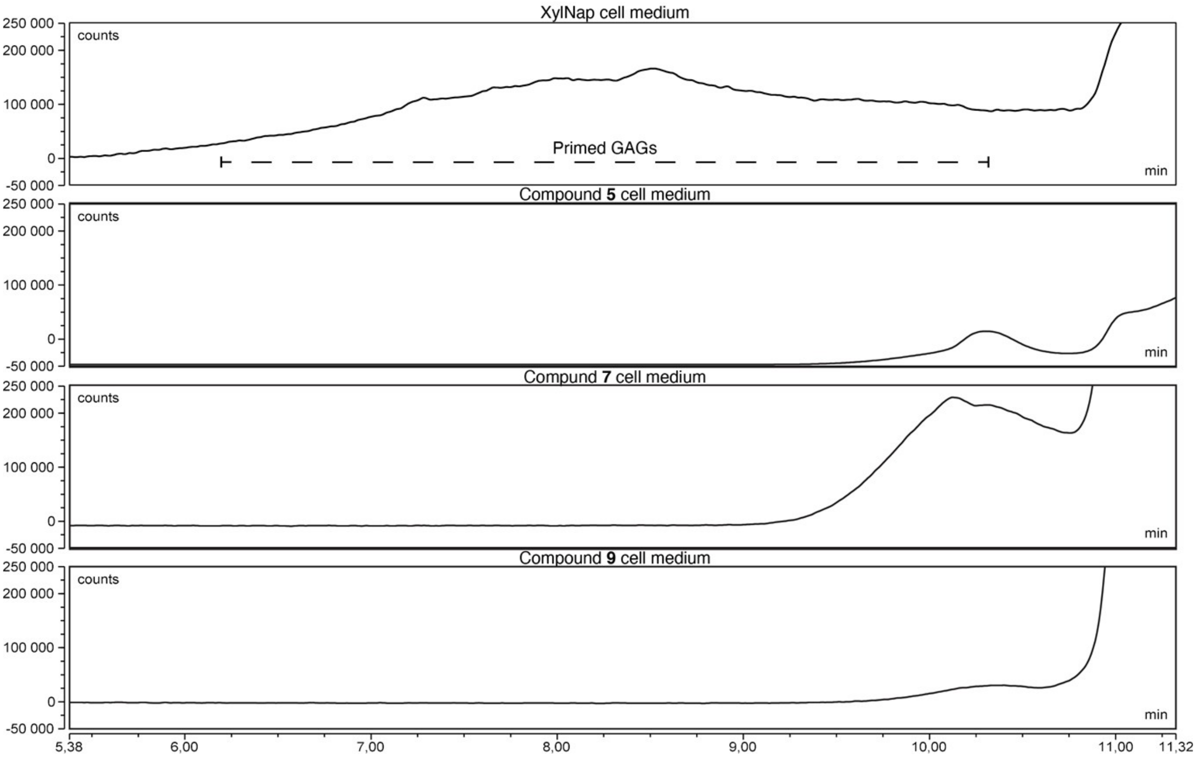

Supplementary Figure 1. Size-exclusion chromatography analysis of conditioned medium from A549 cells treated with 0.1 mM **XylNap**, **5**, **7** or **9**. Fluorescence was monitored at 229/372 (Ex./Em.) for XylNap and 410/455 (Ex./Em.) for PacBlue compounds.

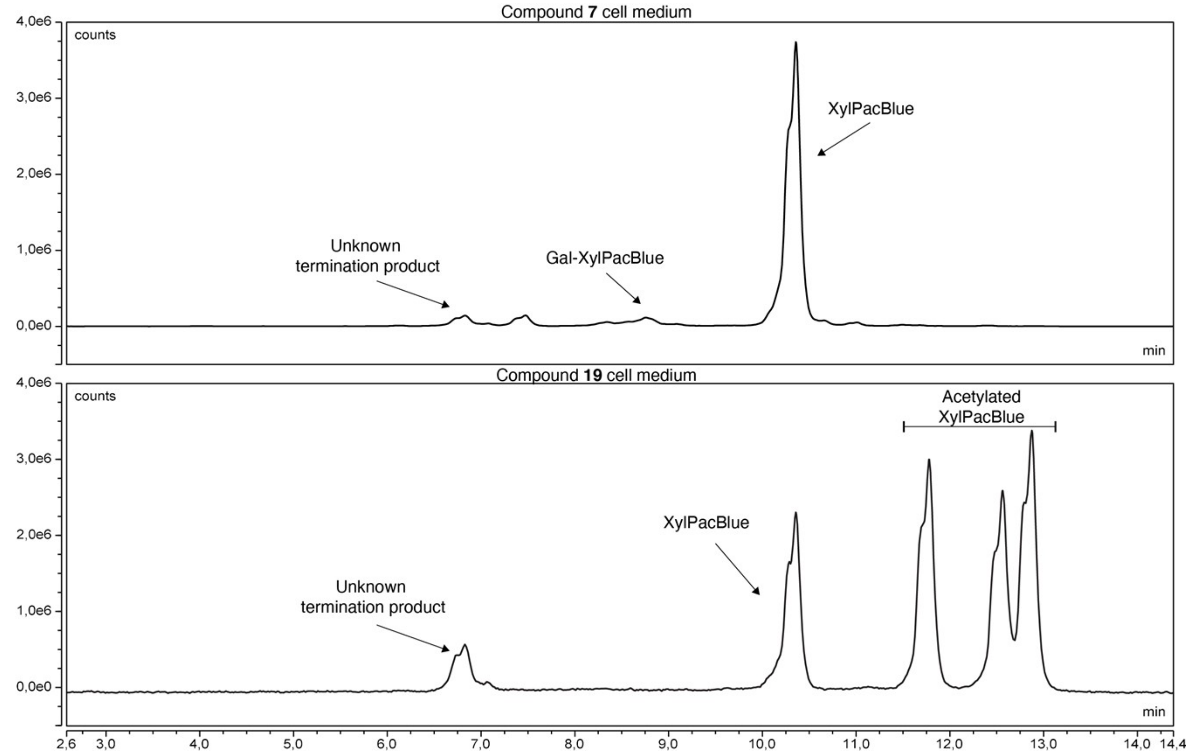

Supplementary Figure 2. Reversed-phase chromatography analysis of products secreted by A549 cells after stimulation with 0.1 mM non-acetylated (top, **7**) or fully acetylated (bottom, **19**) compound.

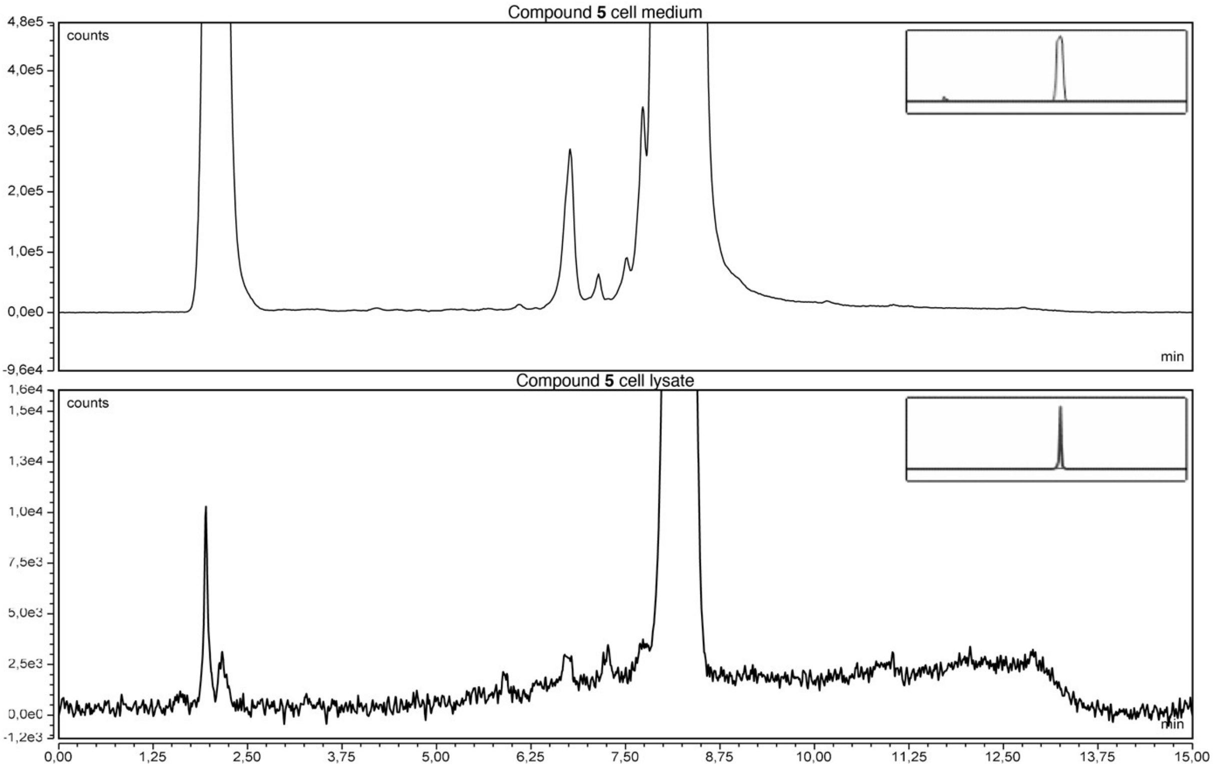

Supplementary Figure 3. Reversed-phase chromatography analysis of conditioned medium and cell lysate from A549 cells treated with 0.1 mM **5.**

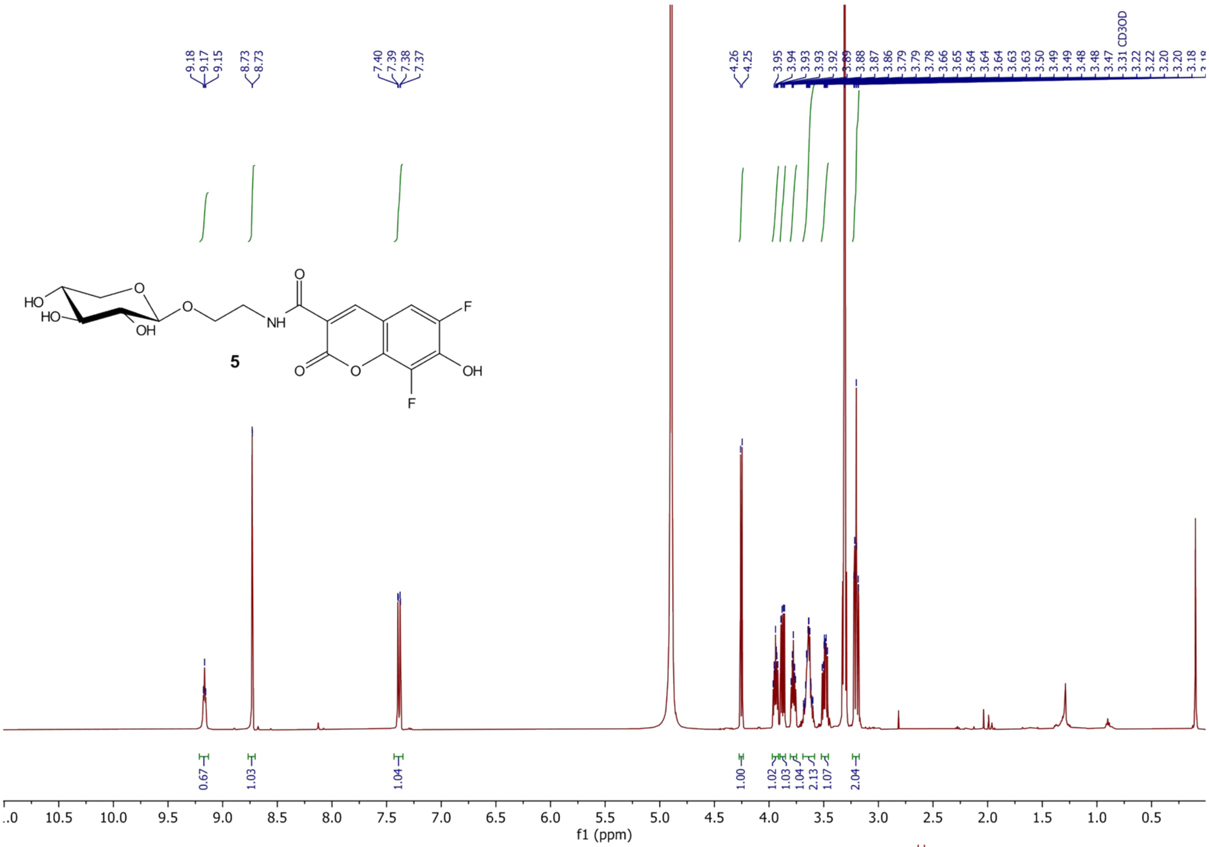

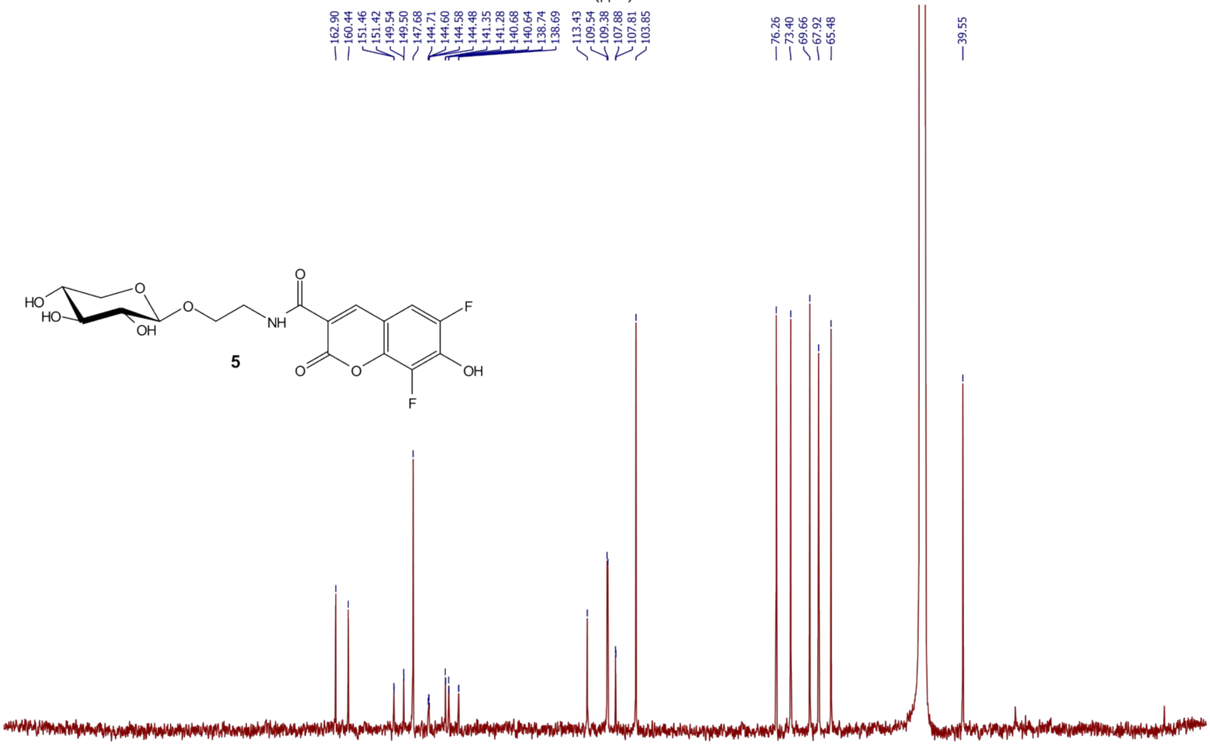

Figure S5 – ^{1}H and ^{13}C spectrum for compound **5**

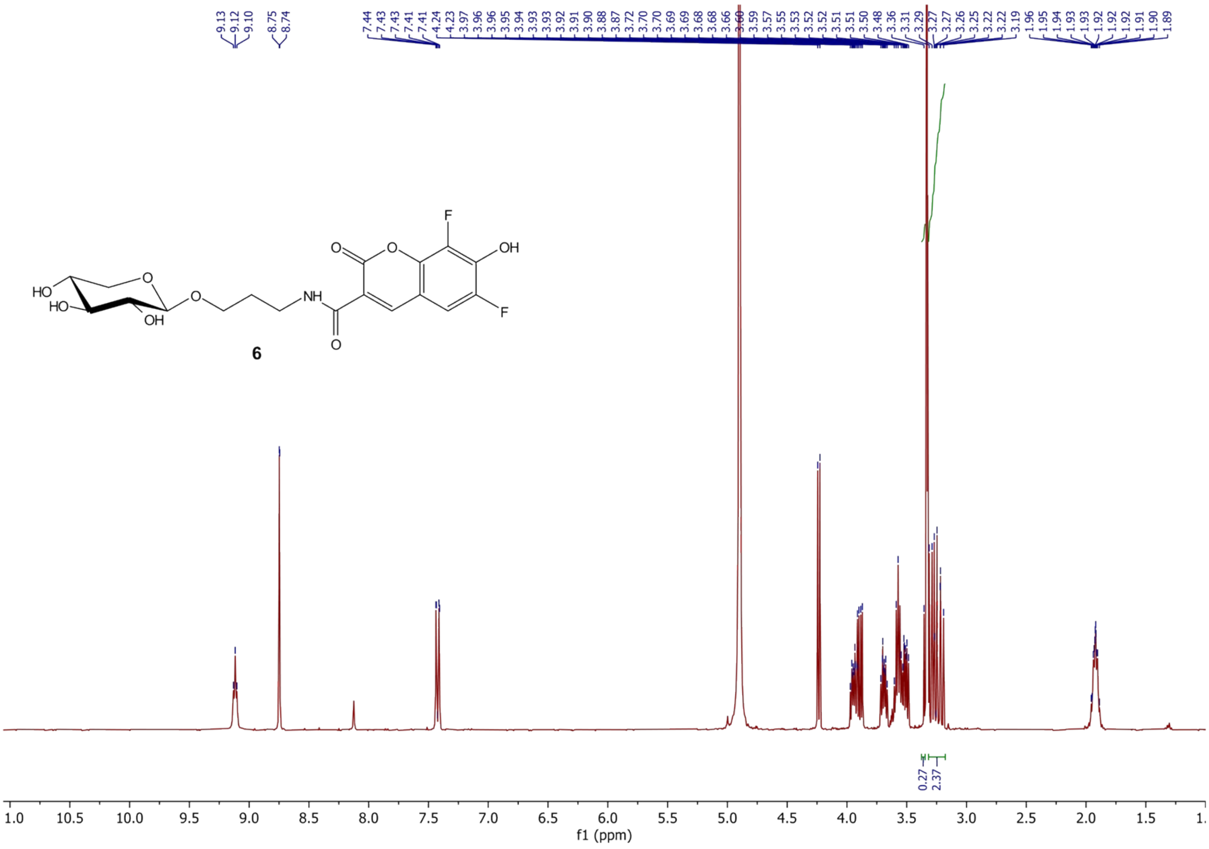

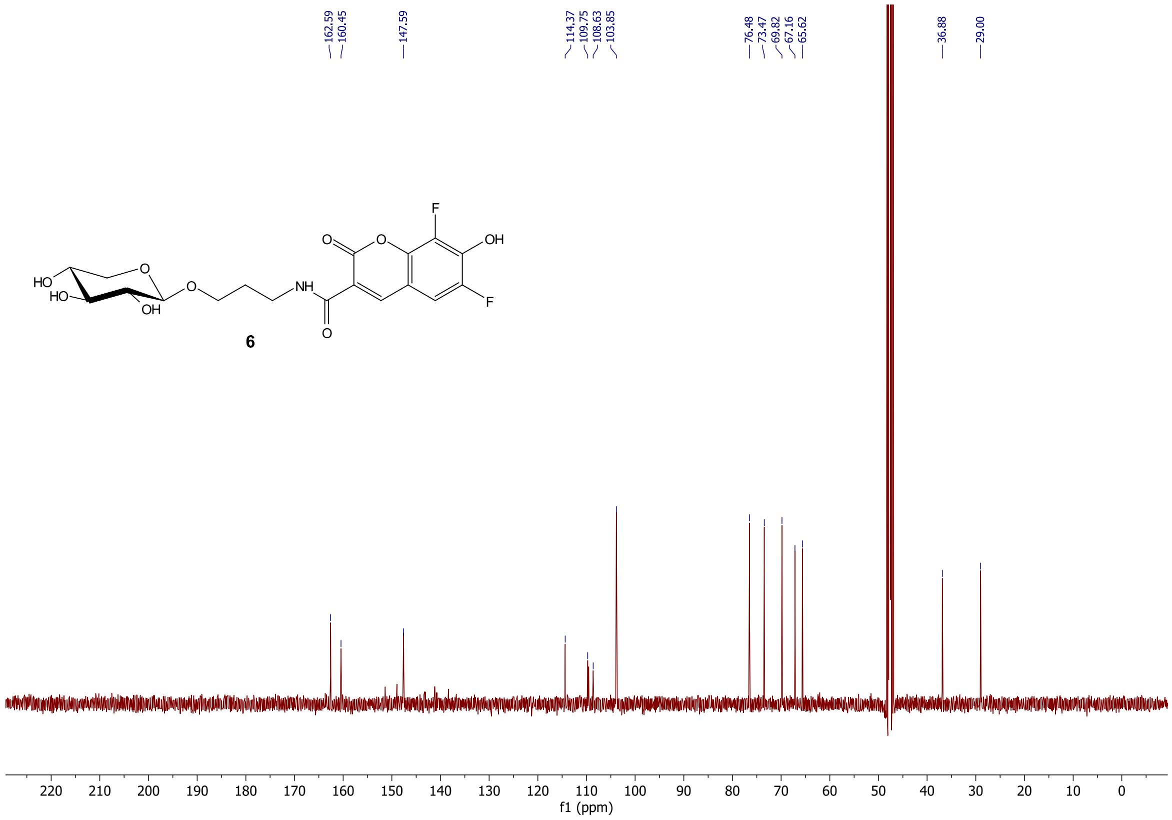

Figure S6 – ^{1}H and ^{13}C spectrum for compound **6**

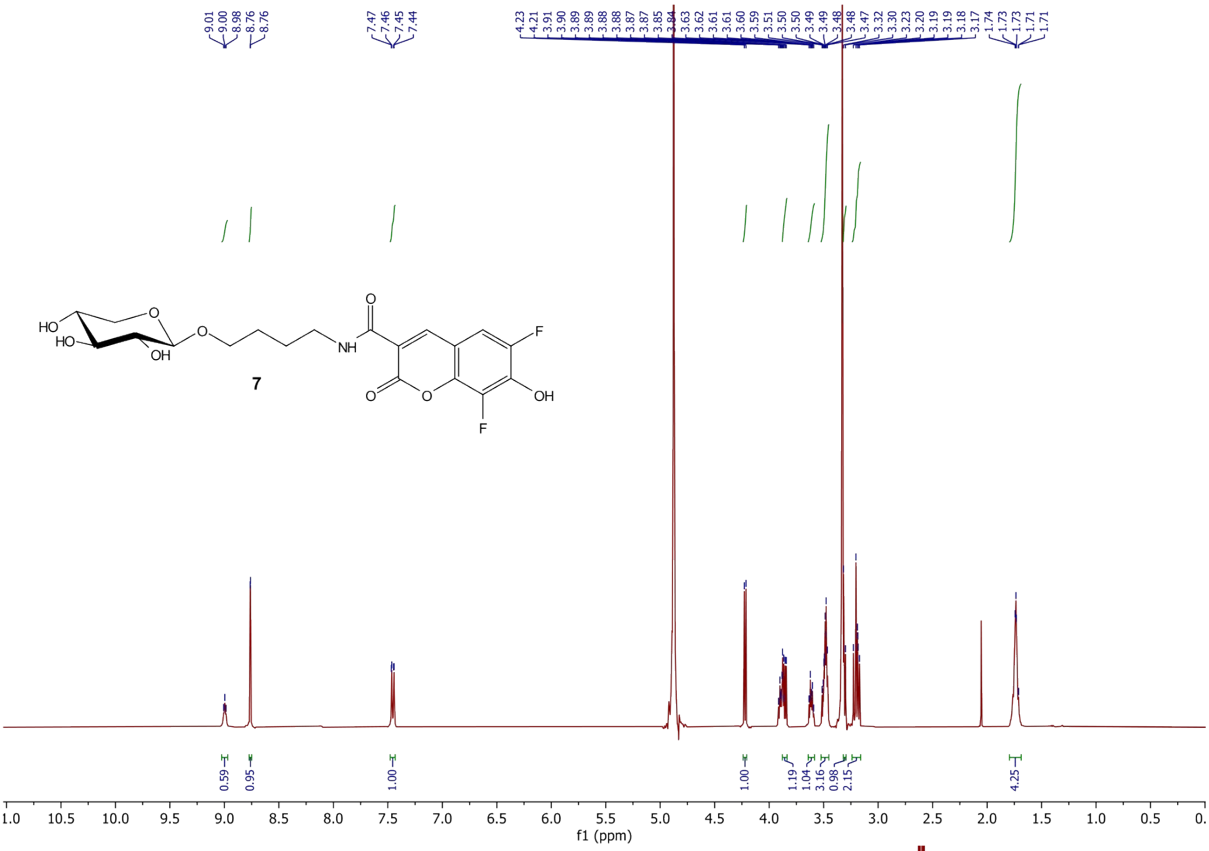

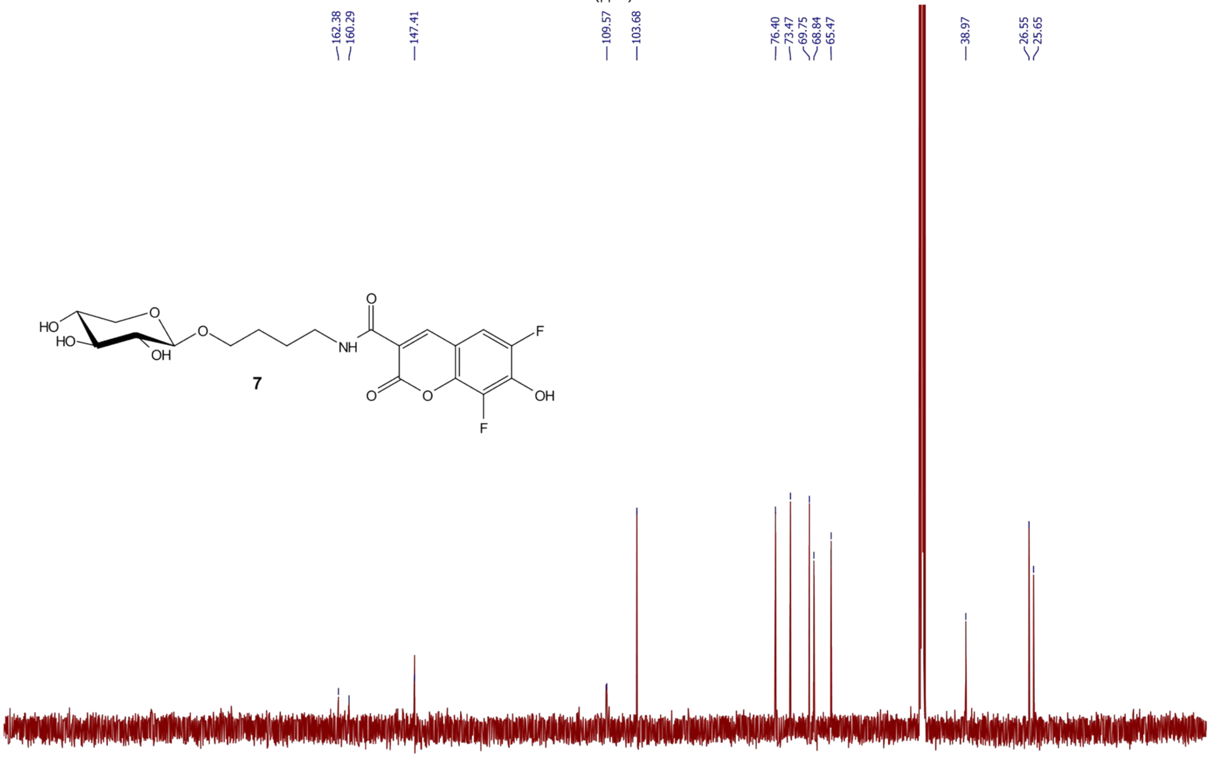

Figure S7 – ^{1}H and ^{13}C spectrum for compound **7**

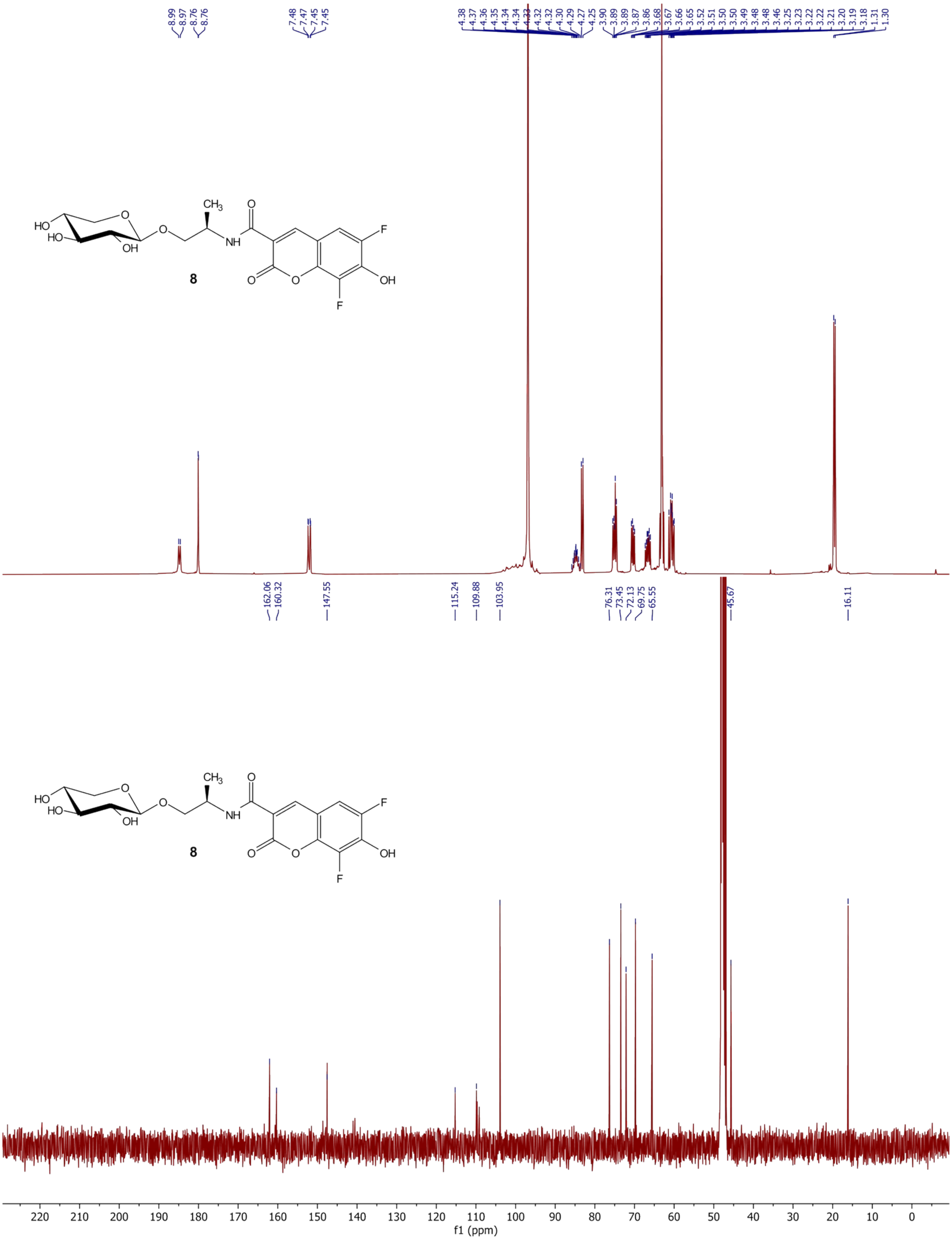

Figure S8 – ^{1}H and ^{13}C spectrum for compound **8**

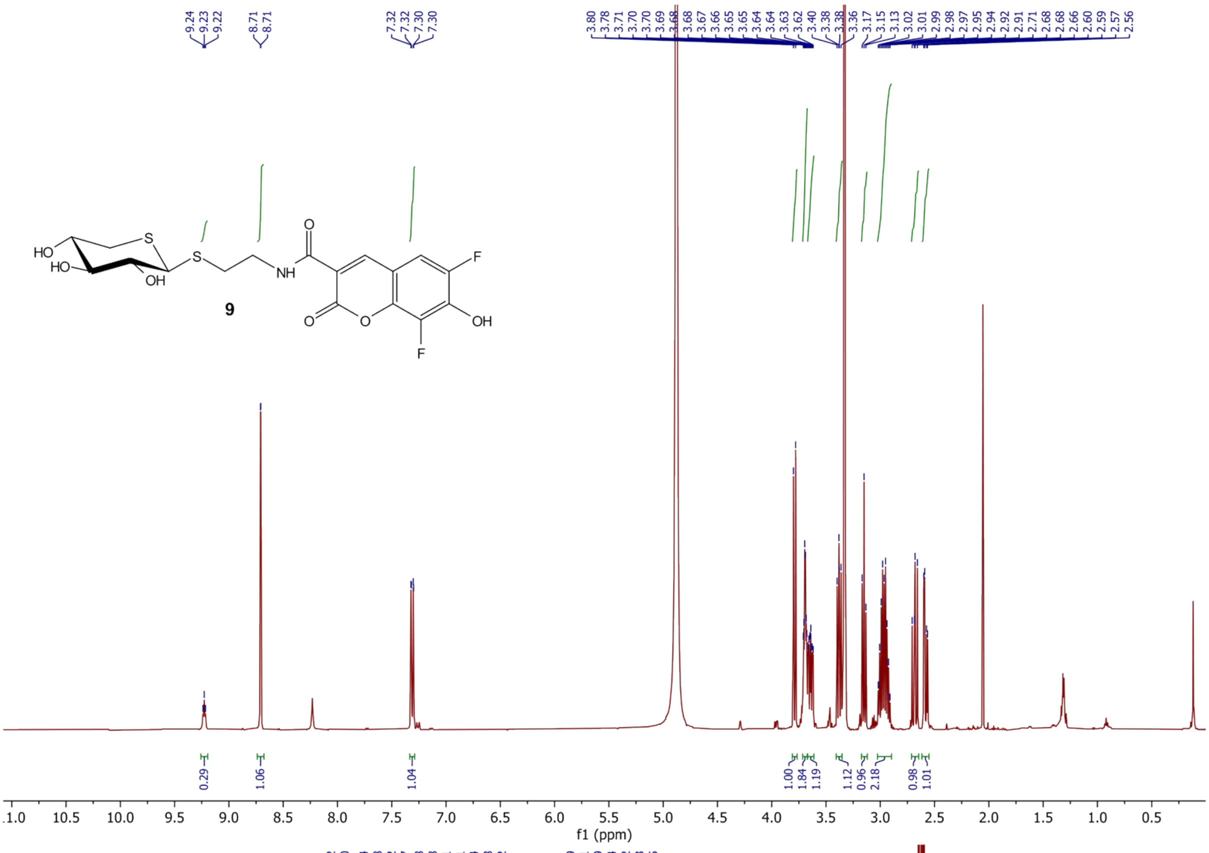

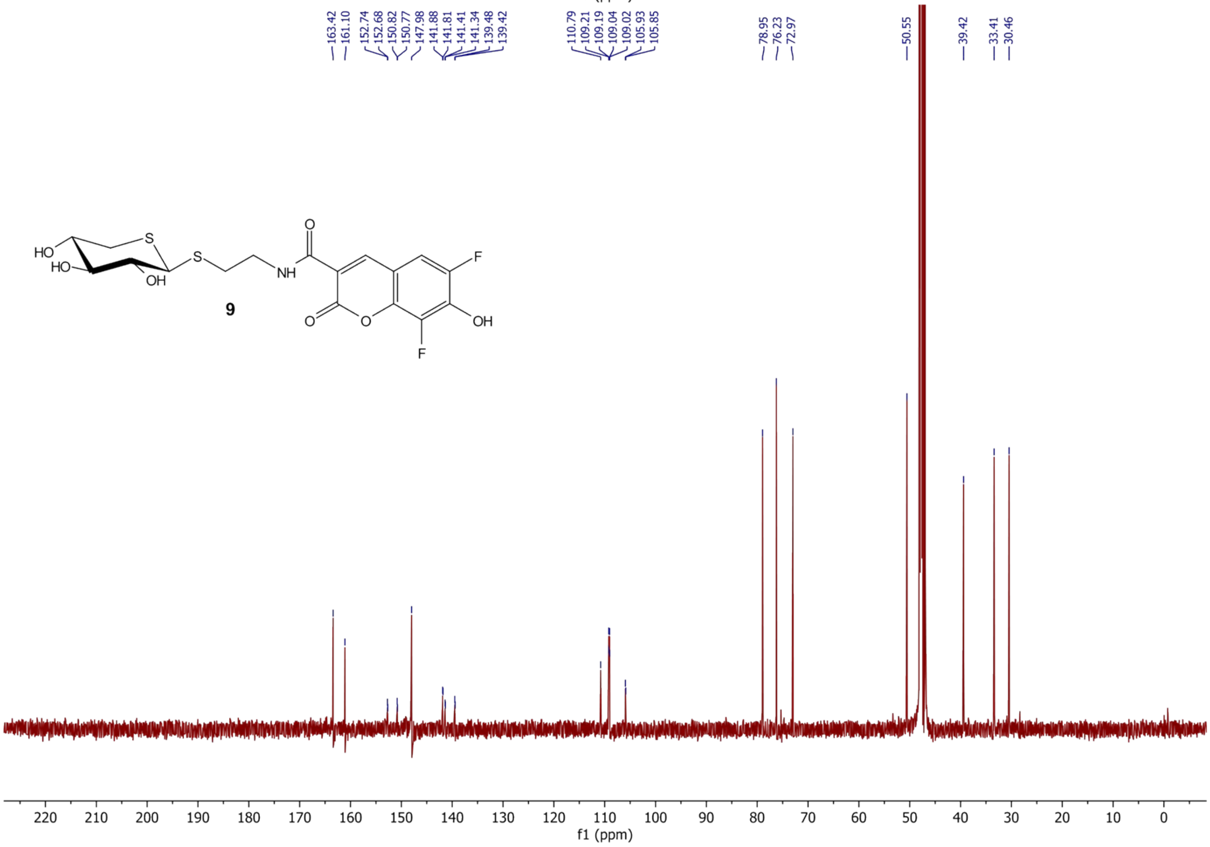

Figure S9 – ^{1}H and ^{13}C spectrum for compound **9**

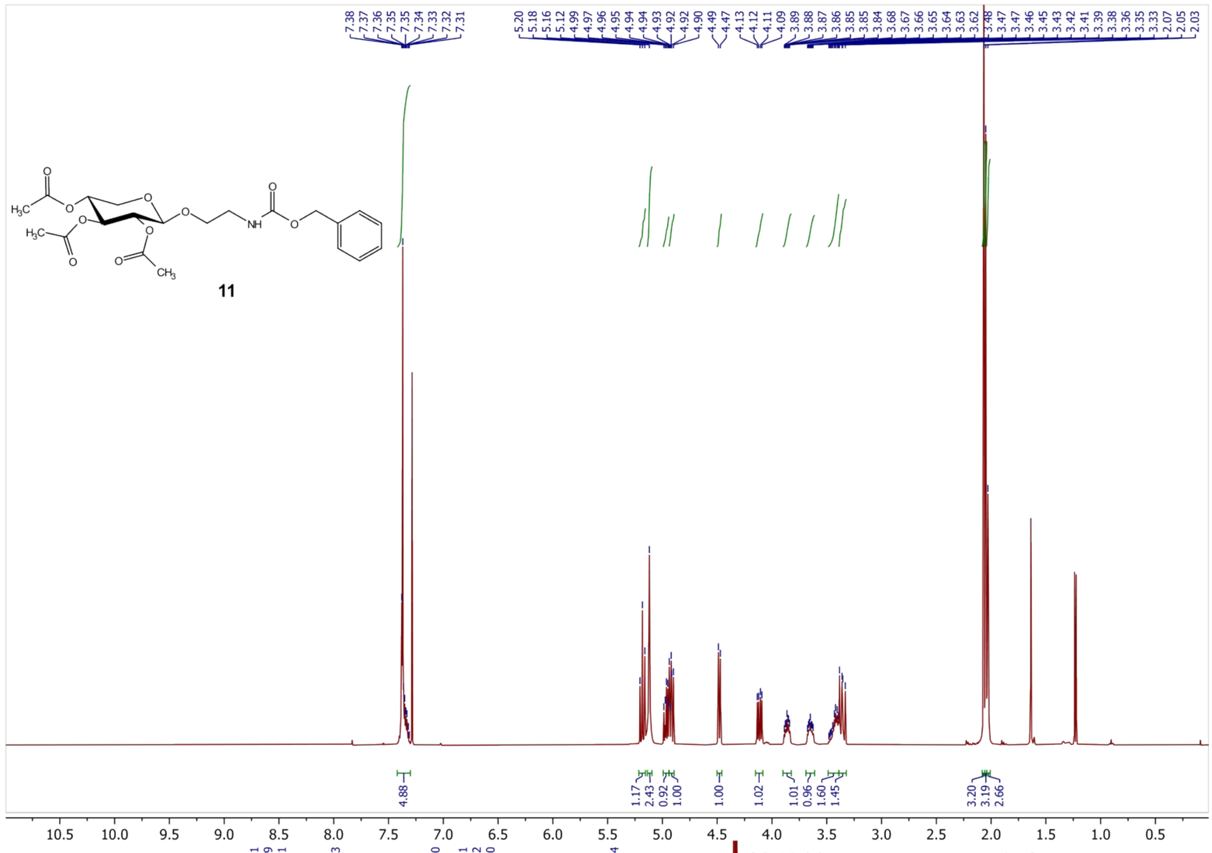

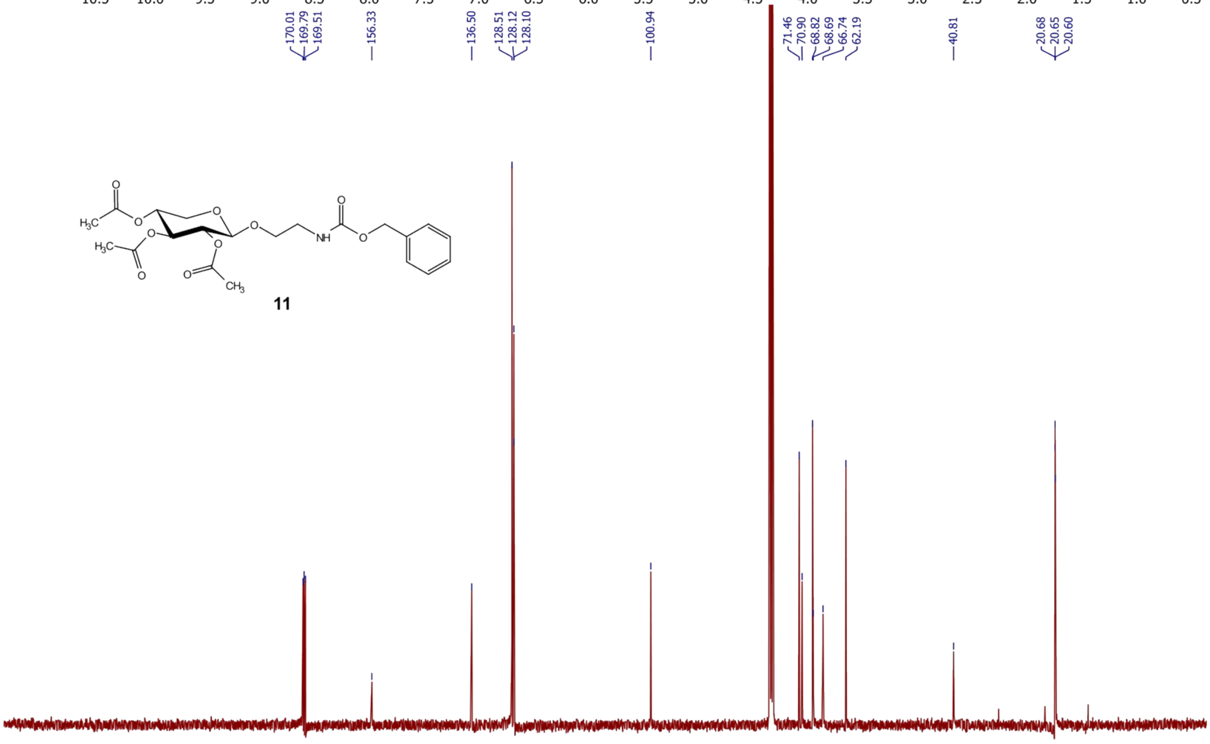

Figure S10 – ^{1}H and ^{13}C spectrum for compound **11**

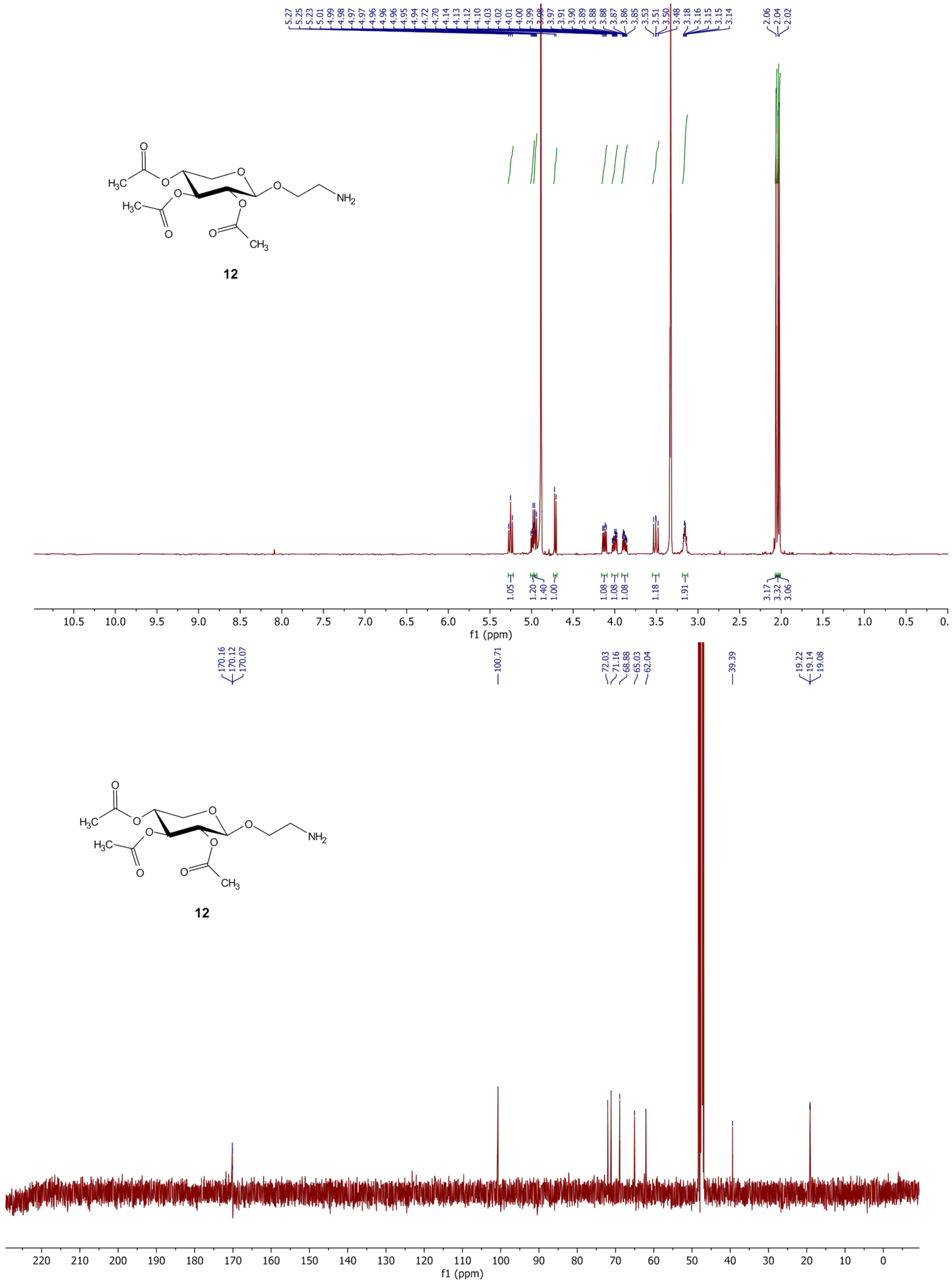

Figure S11 – ^{1}H and ^{13}C spectrum for compound **12**

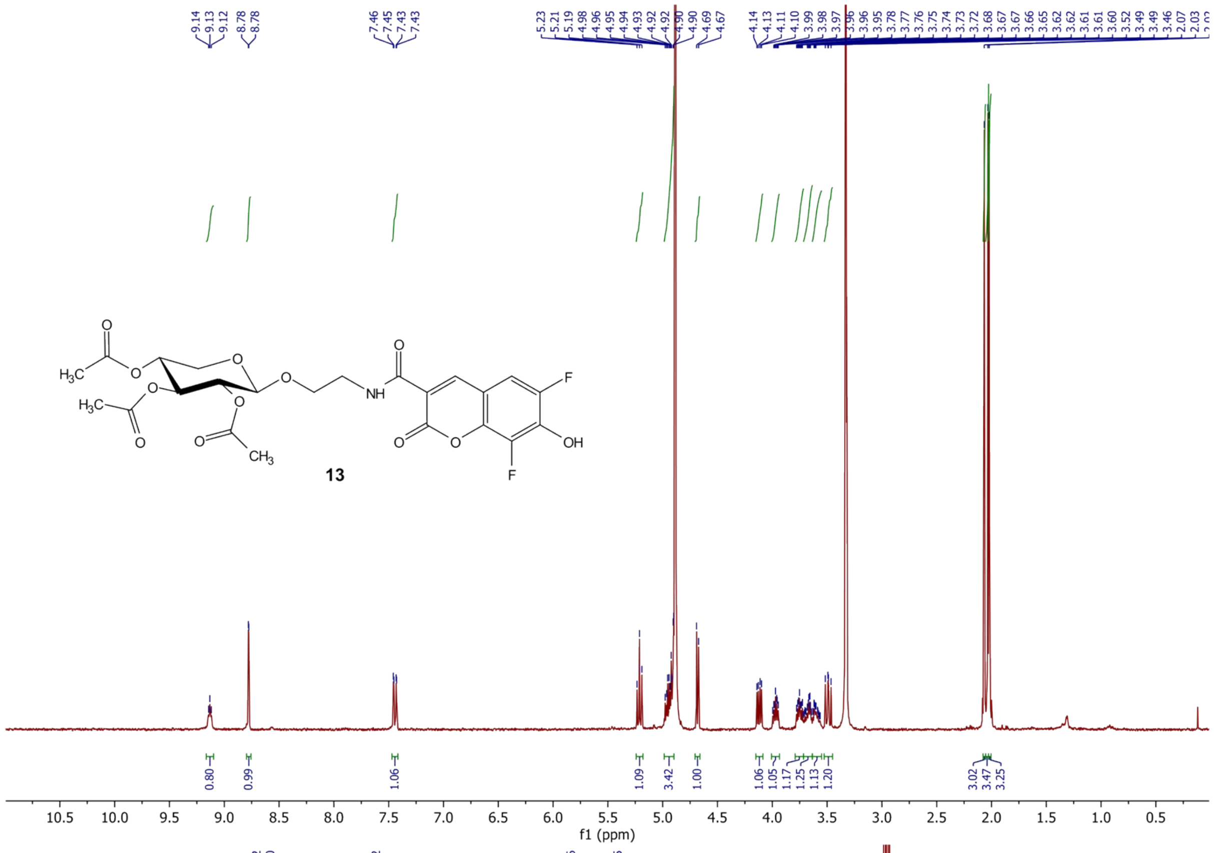

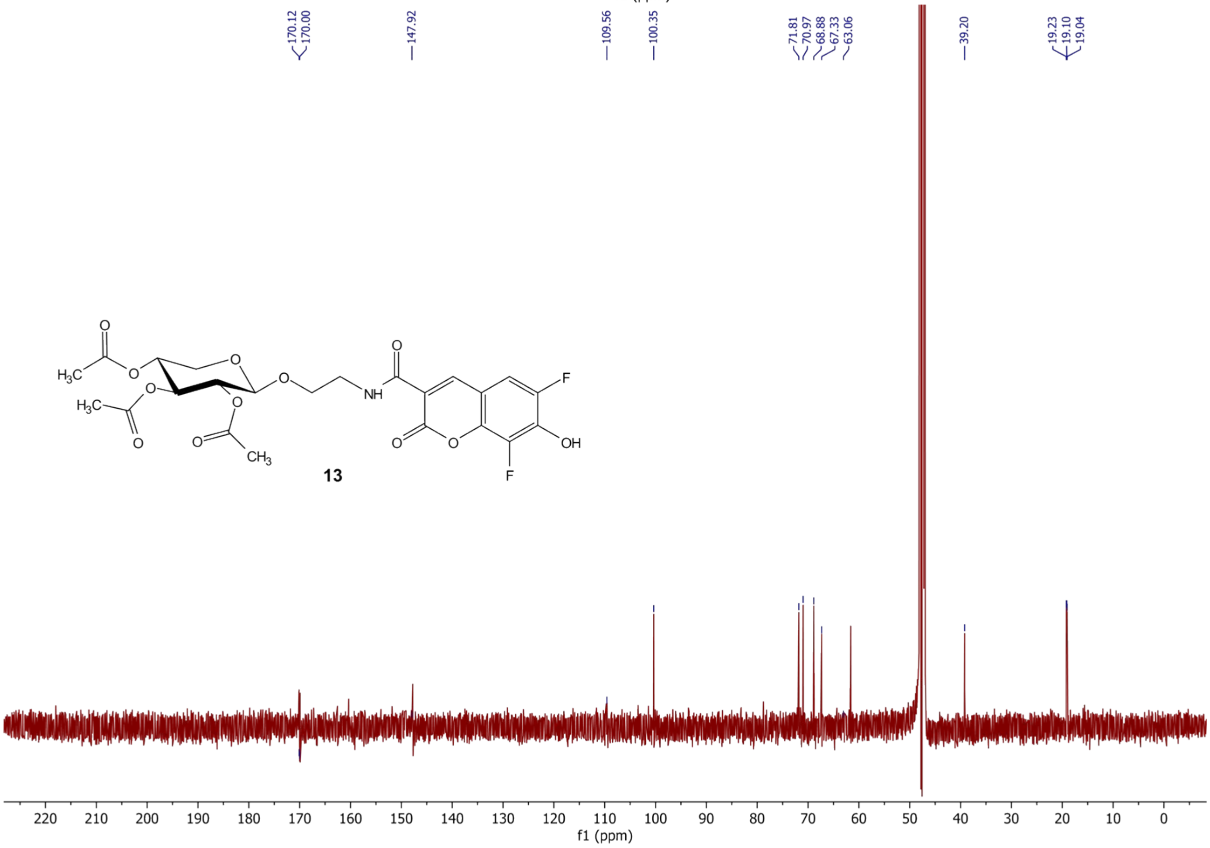

Figure S12 – ^{1}H and ^{13}C spectrum for compound **13**

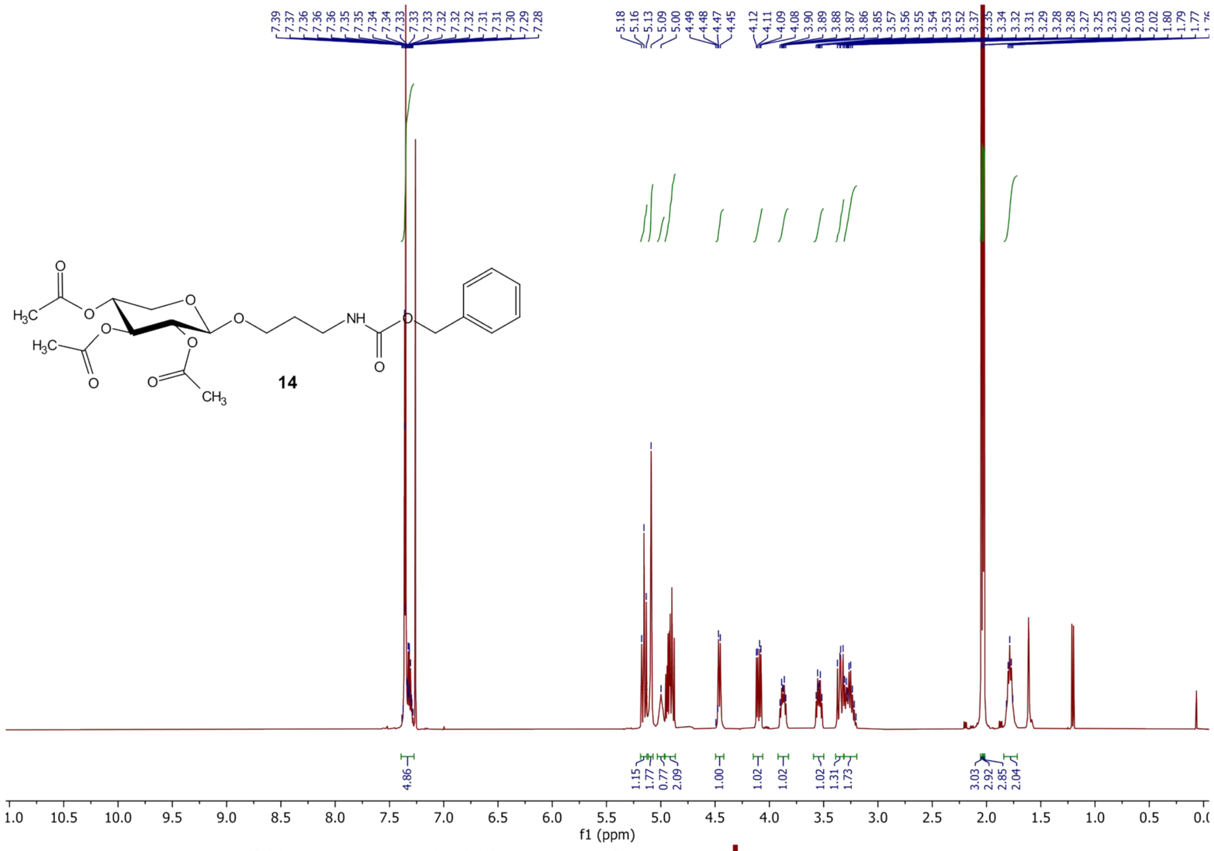

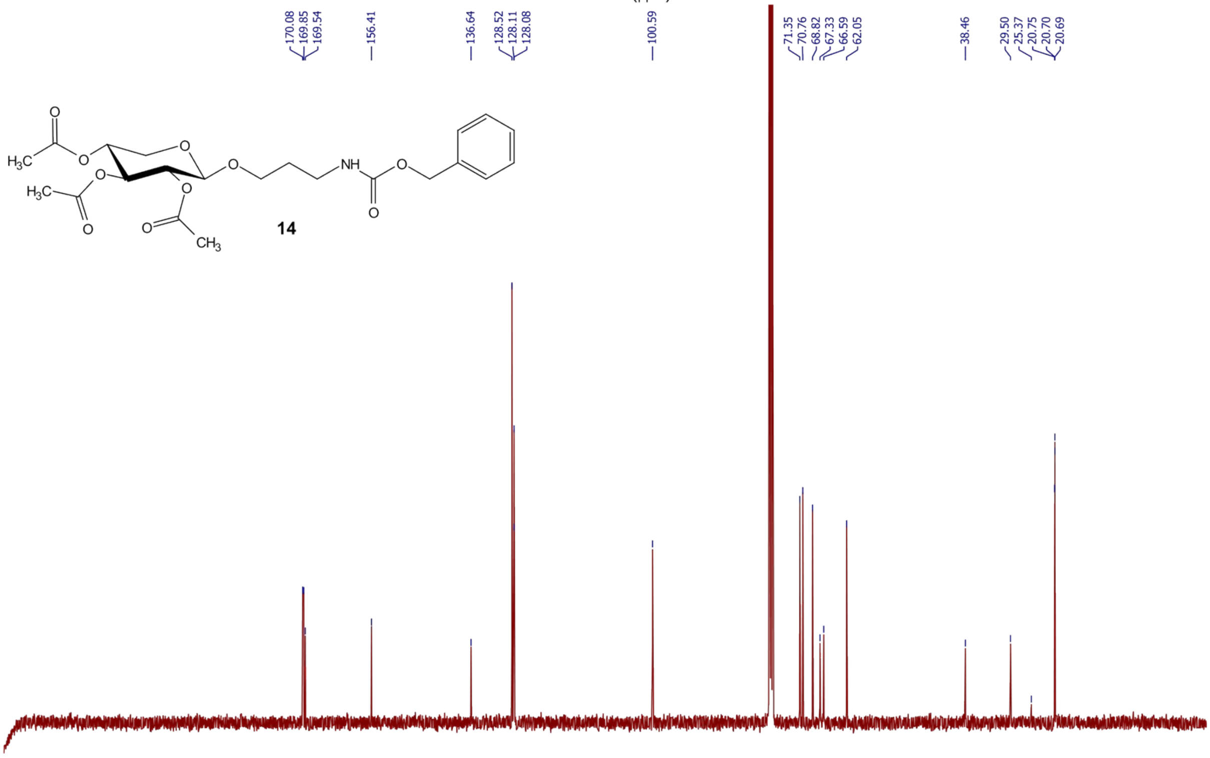

Figure S13 – ^{1}H and ^{13}C spectrum for compound **14**

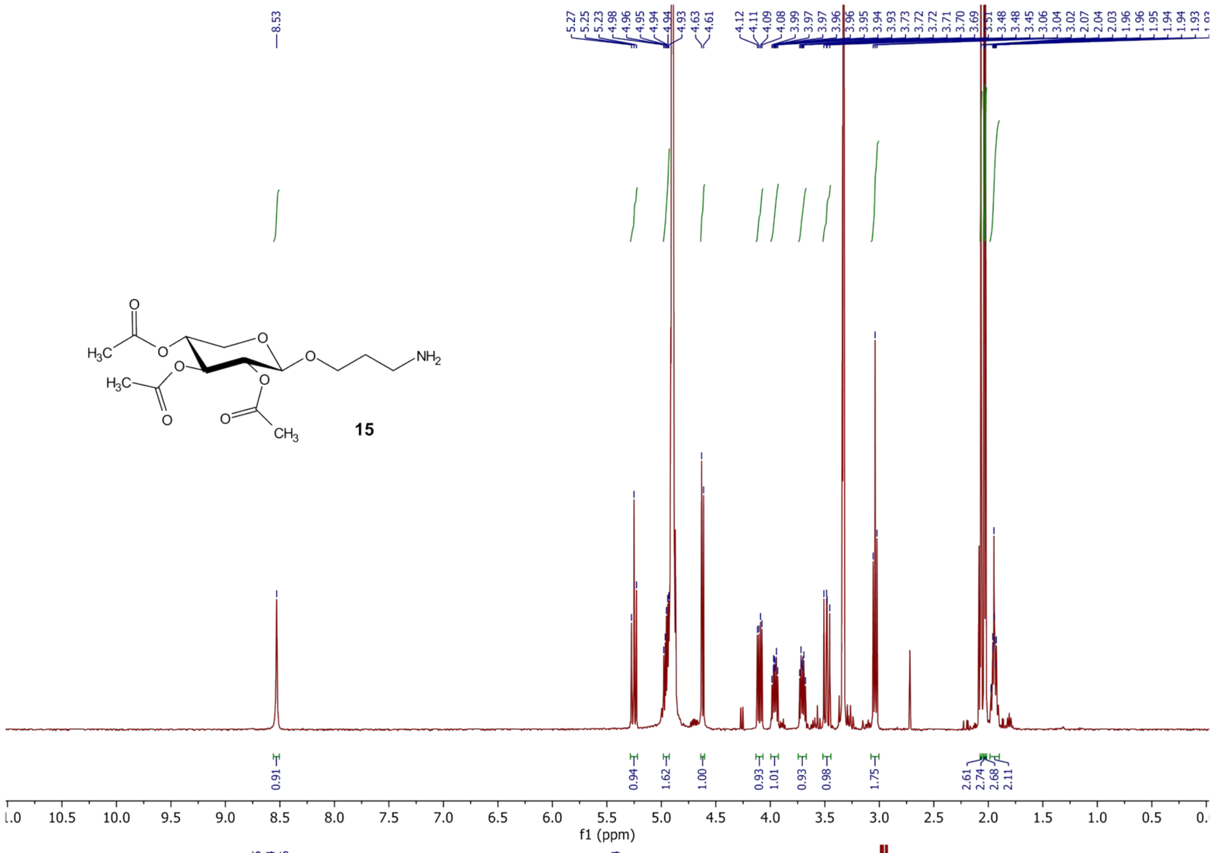

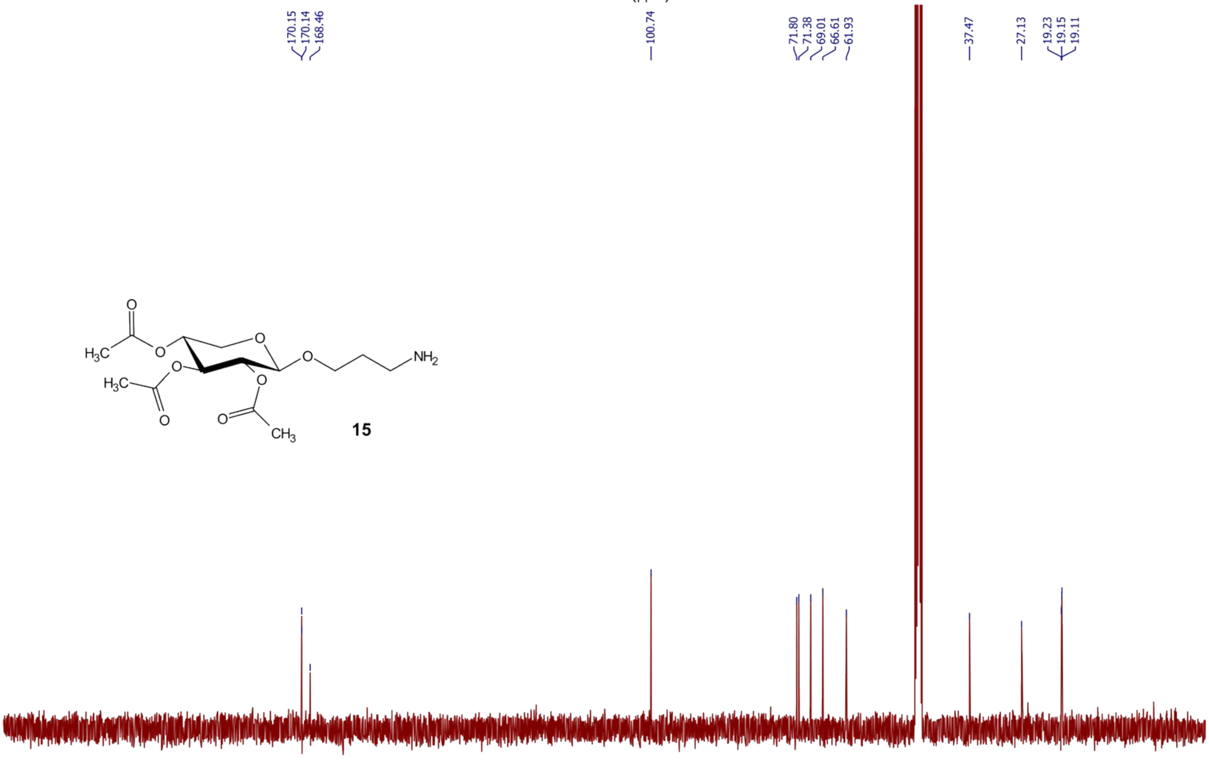

Figure S14 – ^{1}H and ^{13}C spectrum for compound **15**

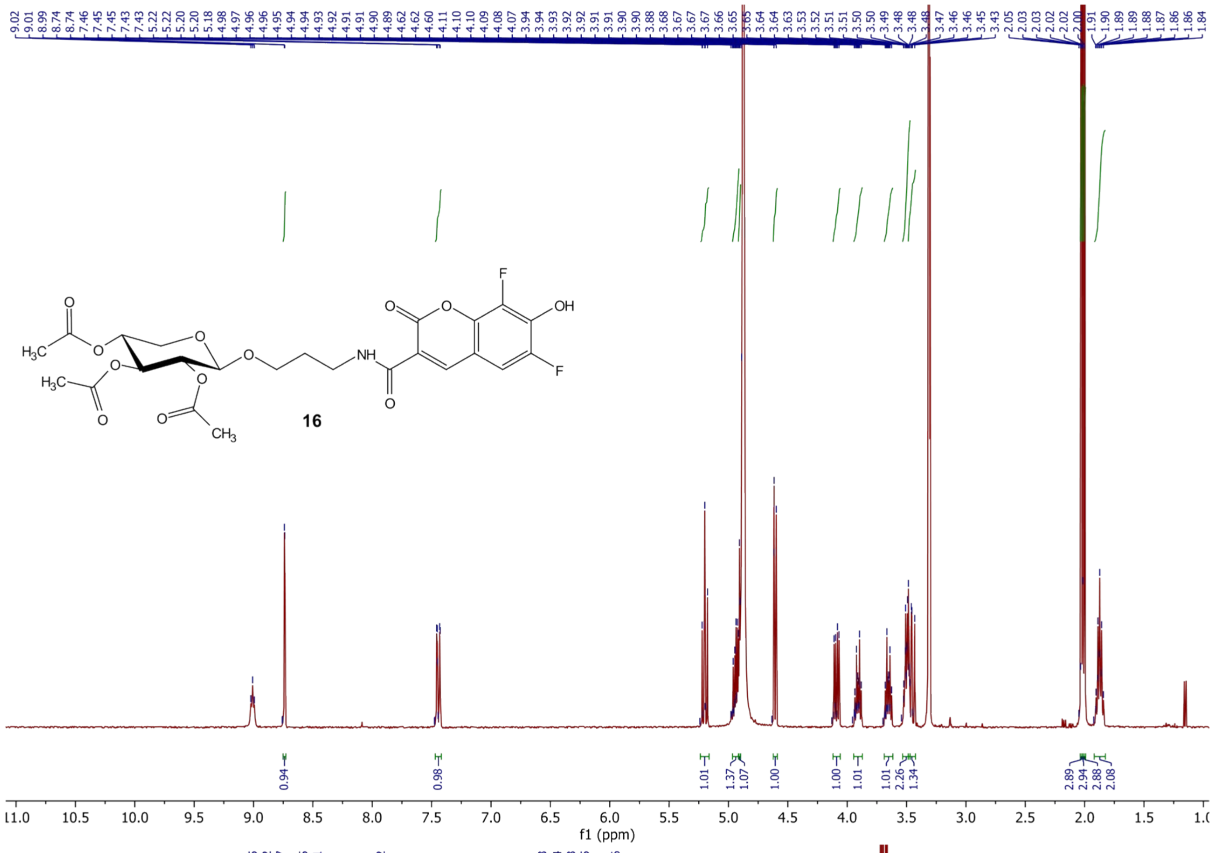

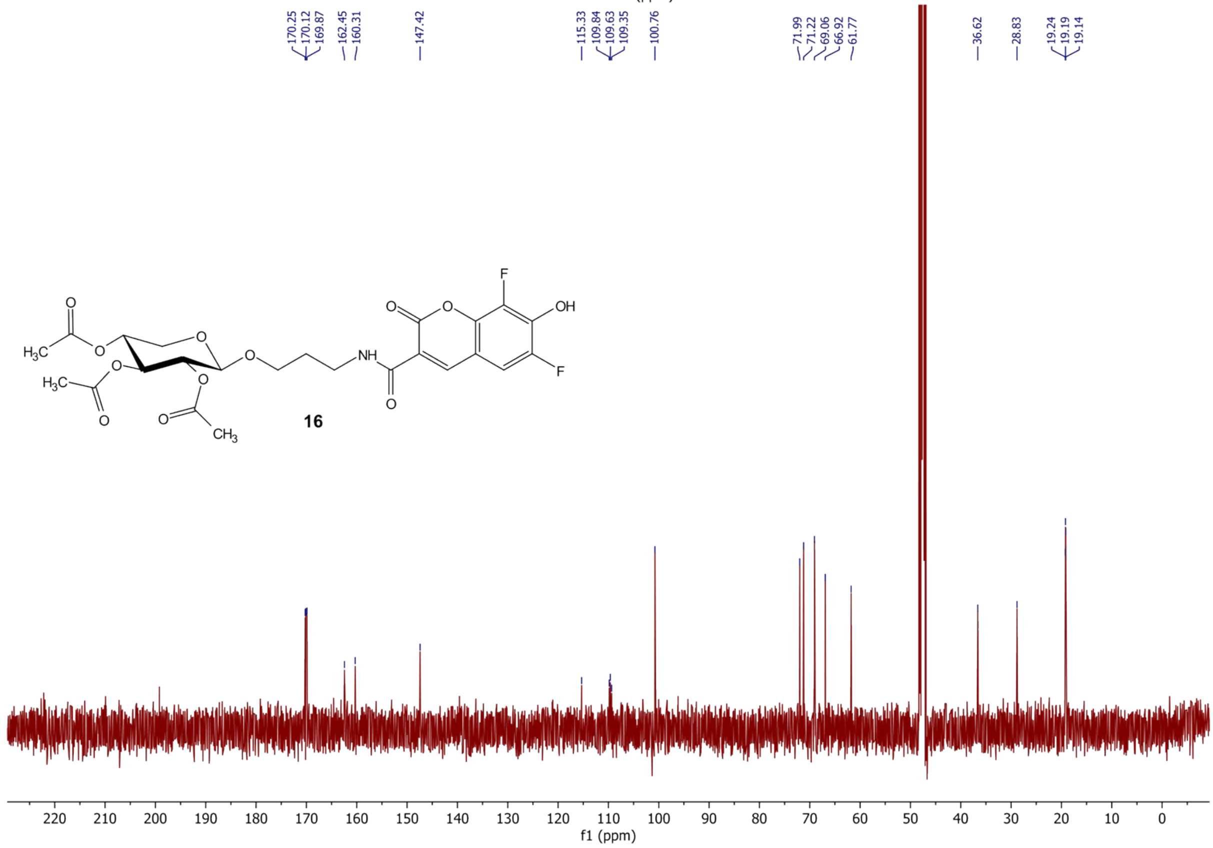

Figure S15 – ^{1}H and ^{13}C spectrum for compound **16**

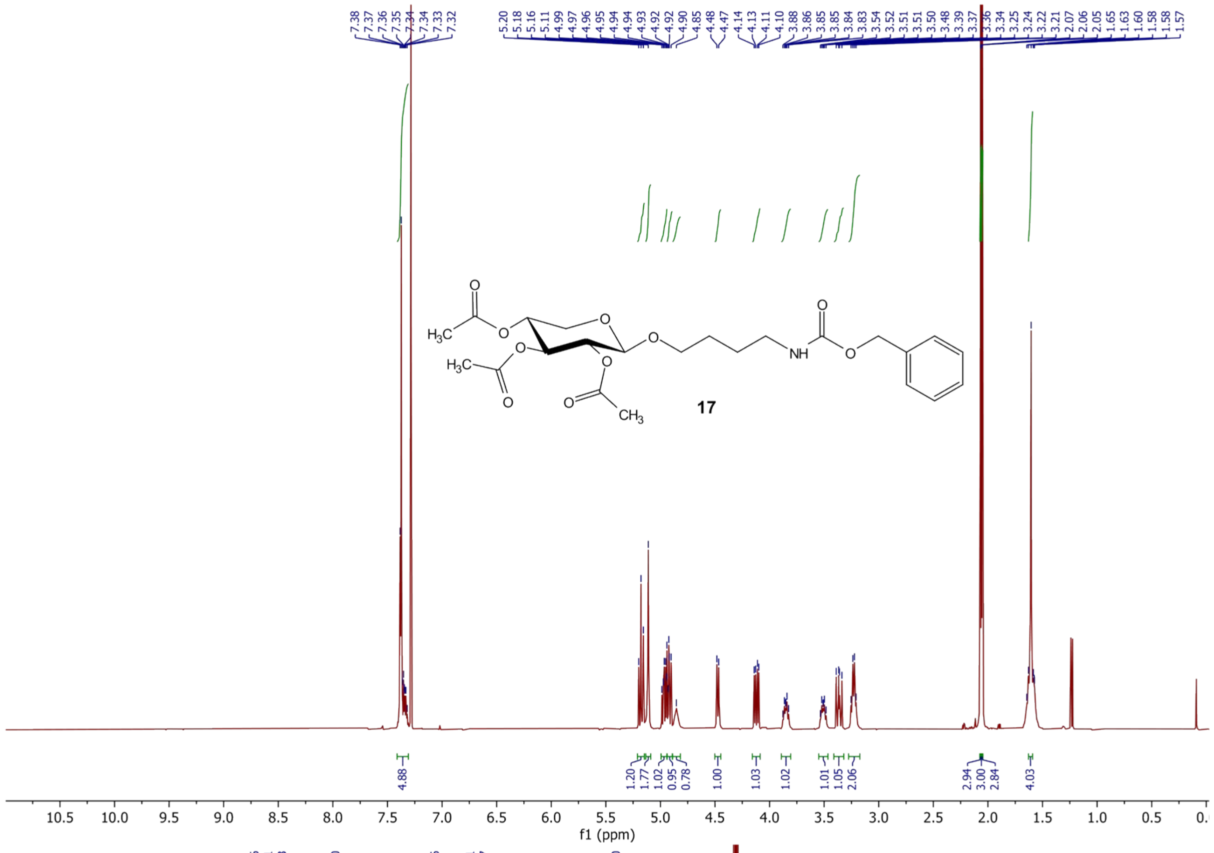

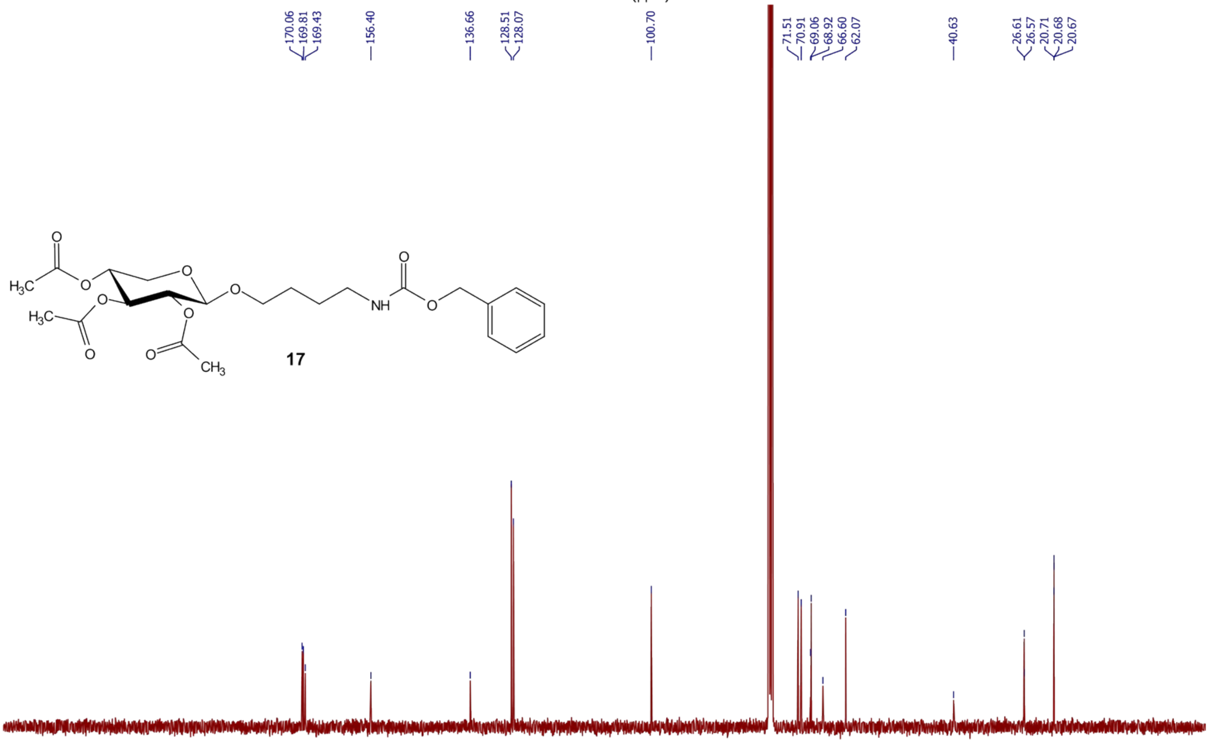

Figure S16 – ^{1}H and ^{13}C spectrum for compound **17**

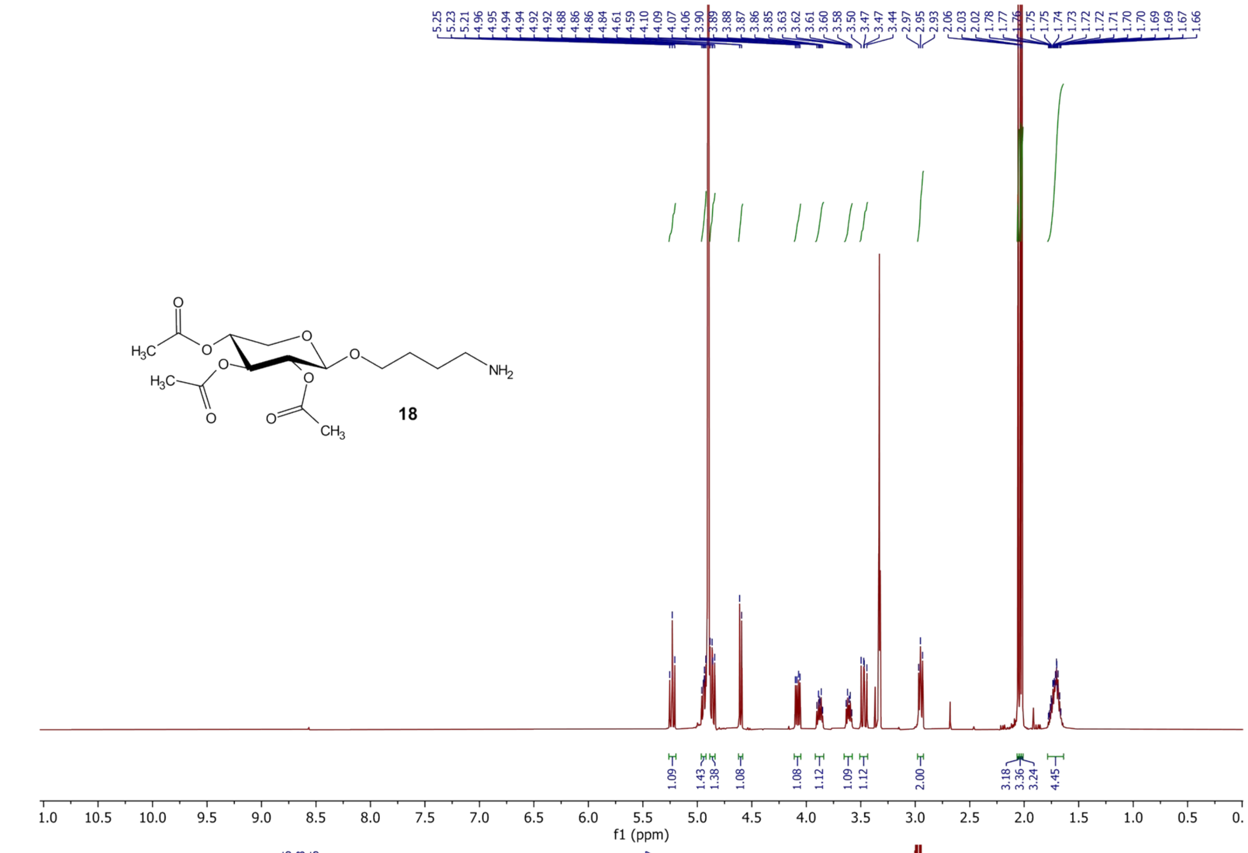

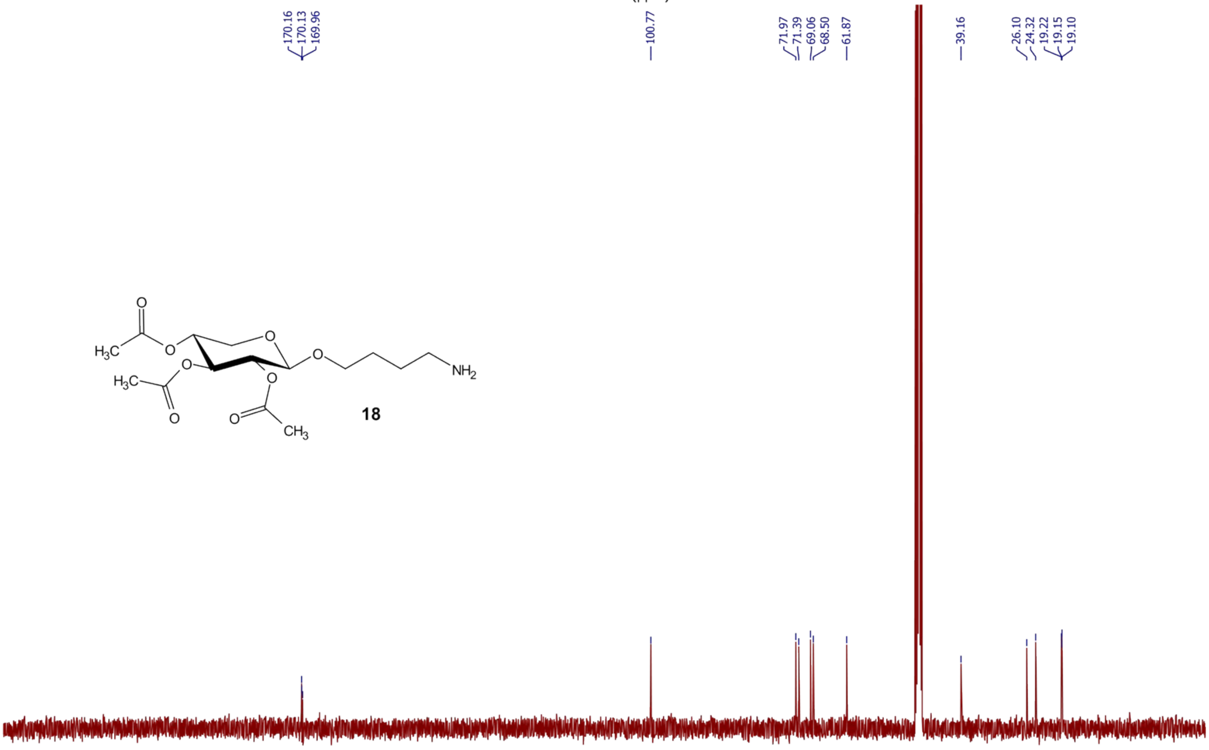

Figure S17– ^{1}H and ^{13}C spectrum for compound **18**

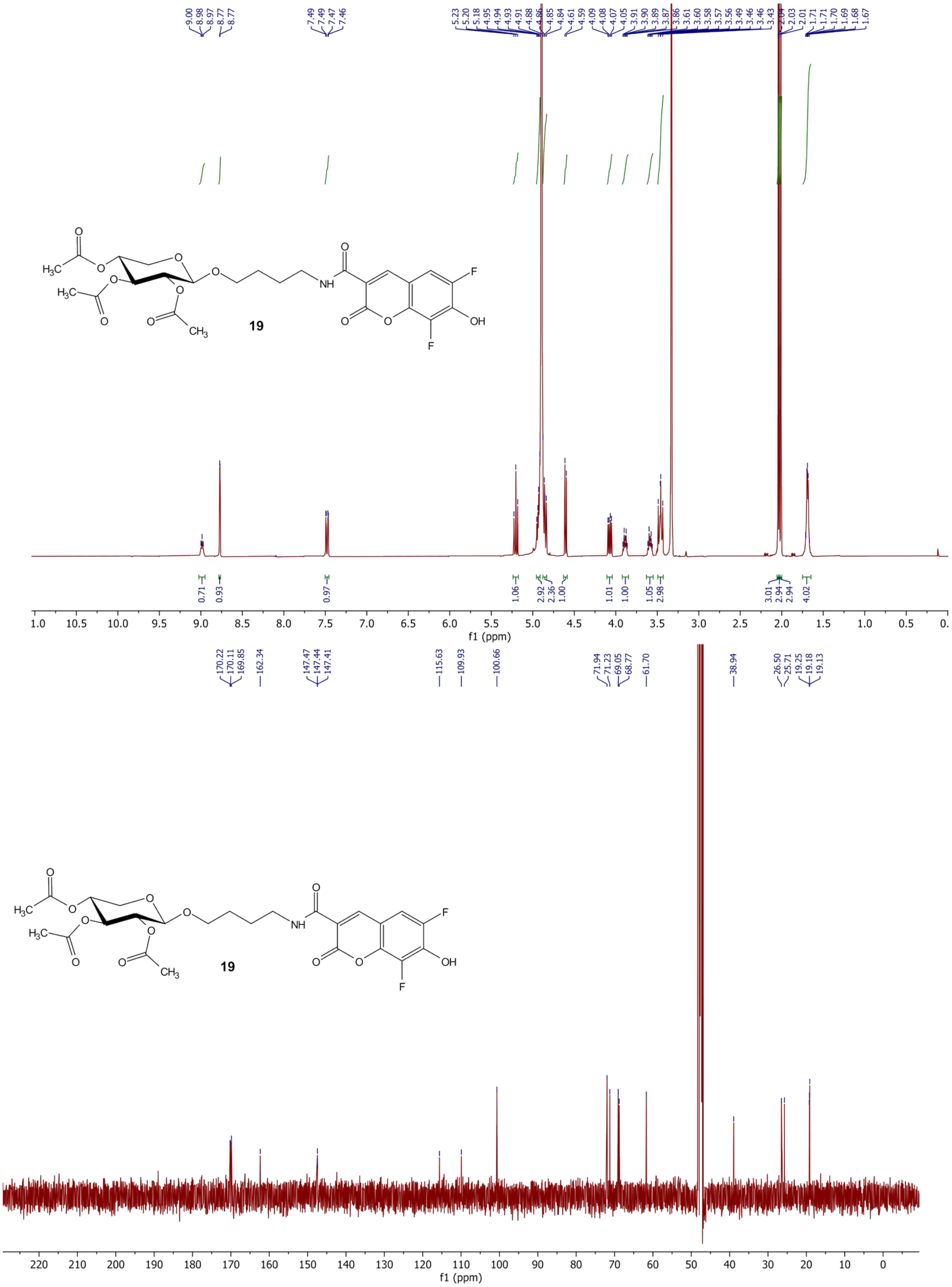

Figure S18– ^{1}H and ^{13}C spectrum for compound **19**

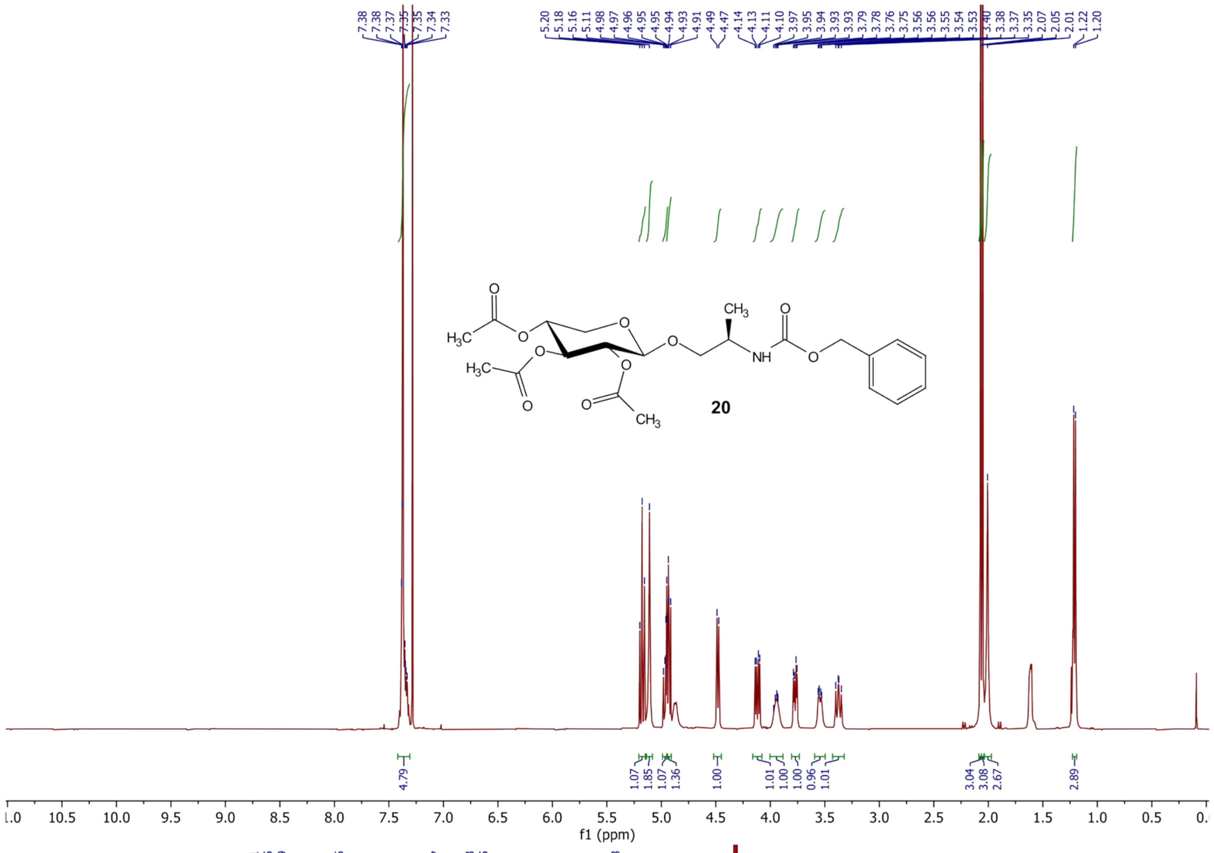

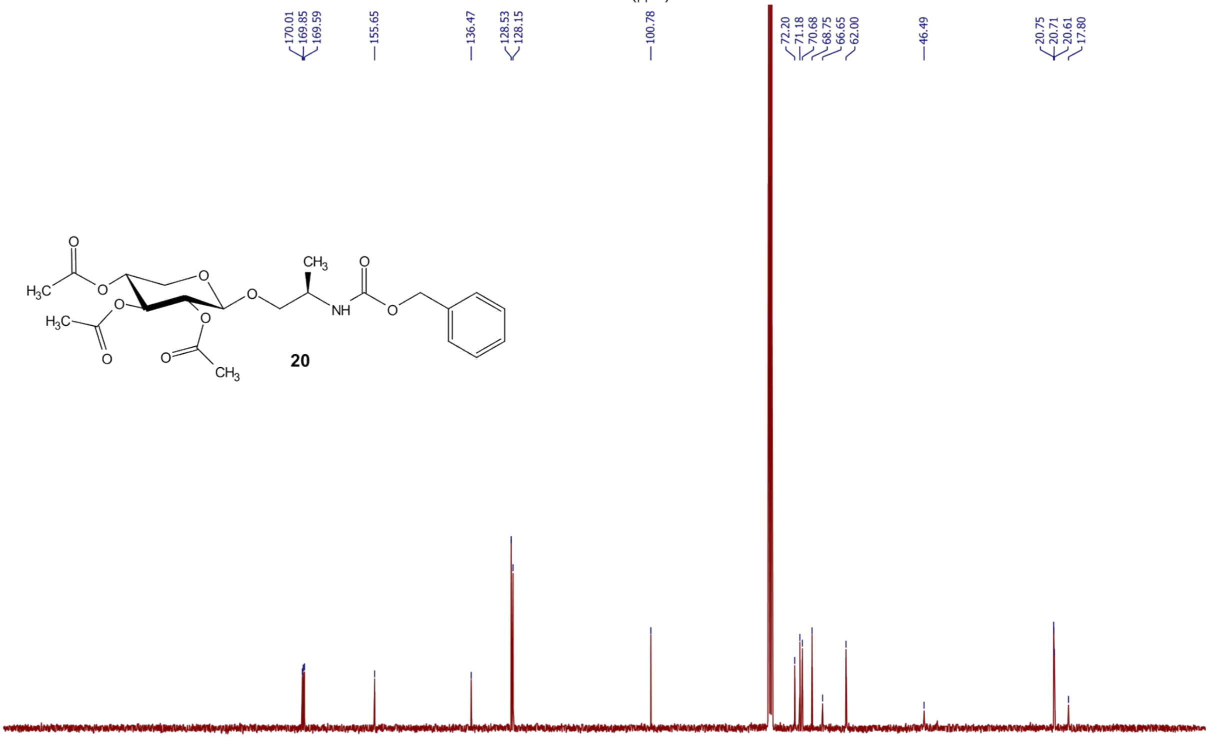

Figure S19 – ^{1}H and ^{13}C spectrum for compound **20**

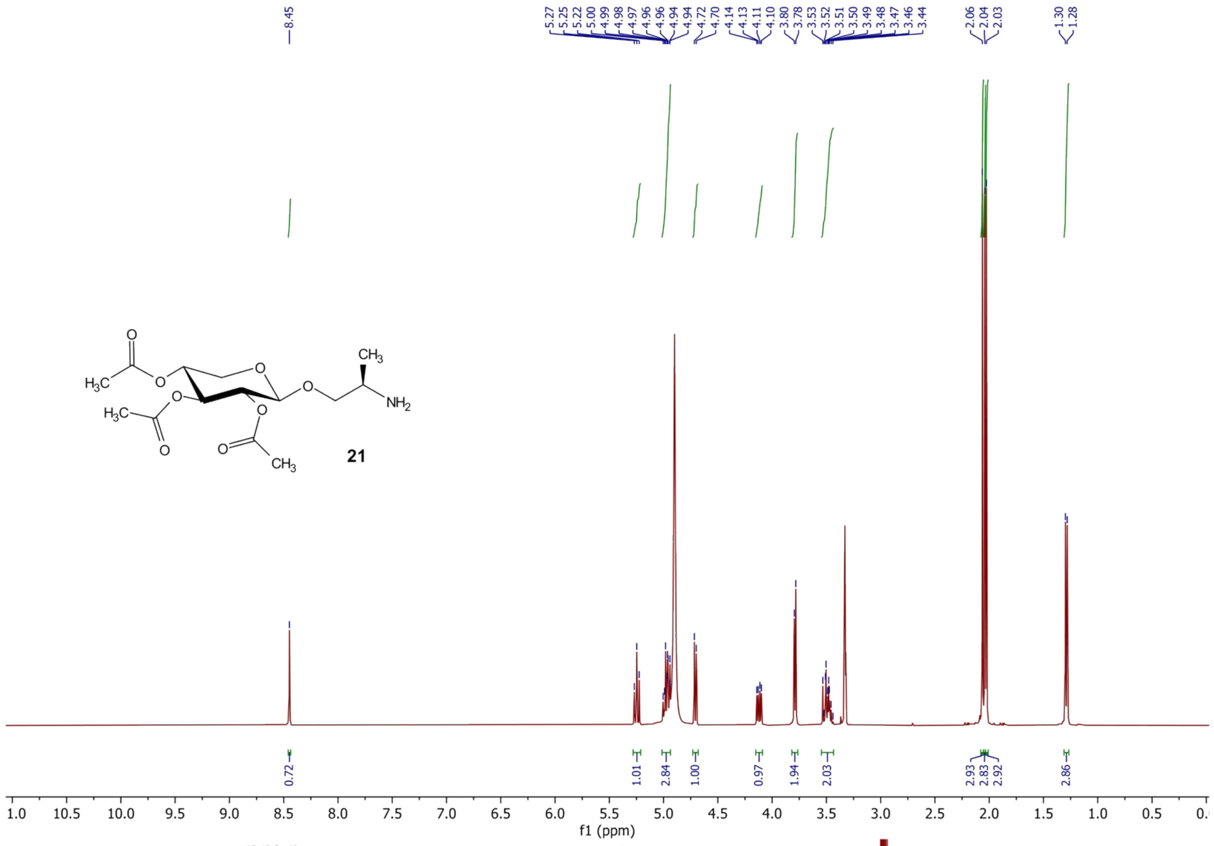

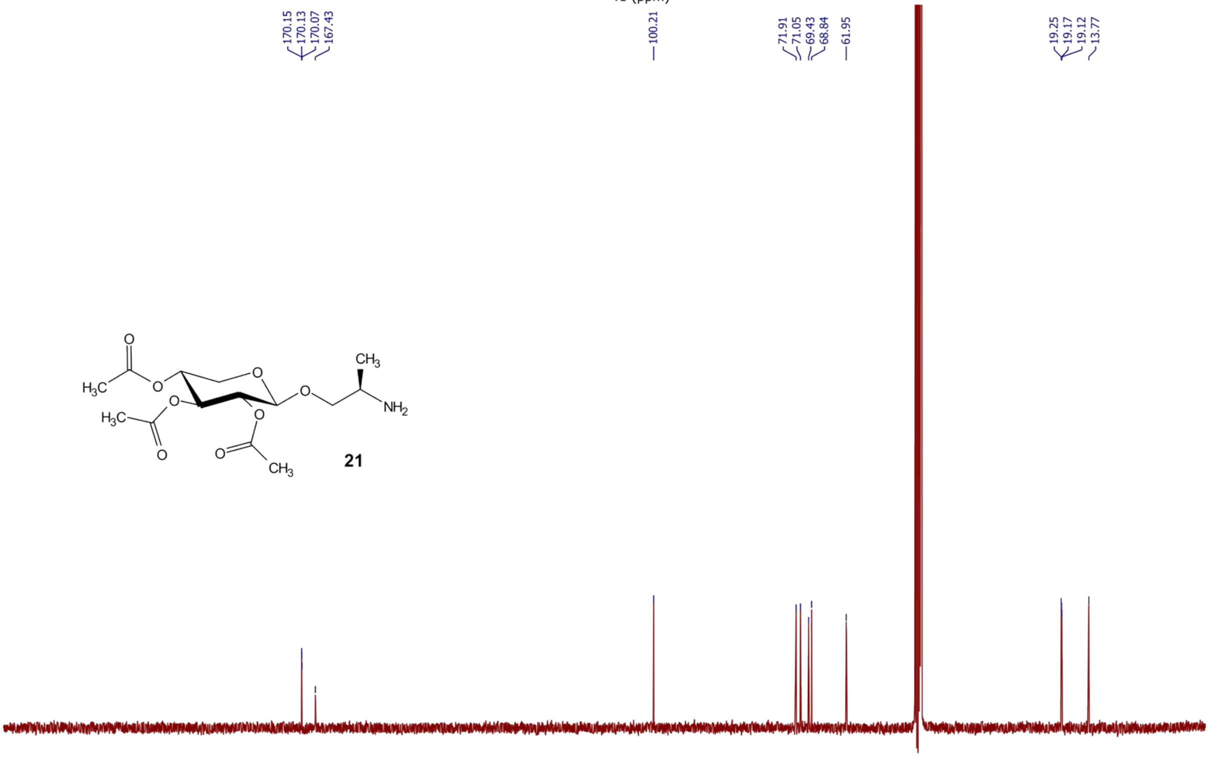

Figure S20 – ¹H and ¹³C spectrum for compound **21**

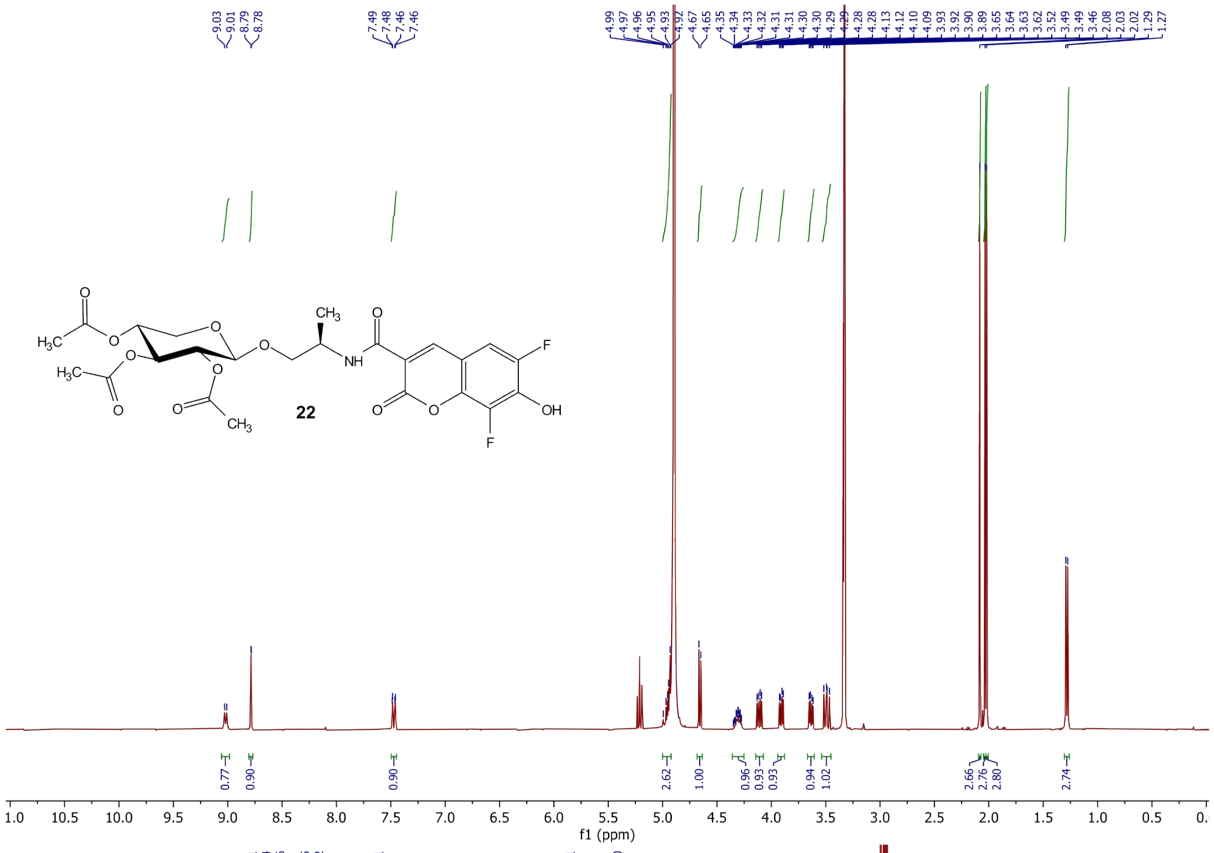

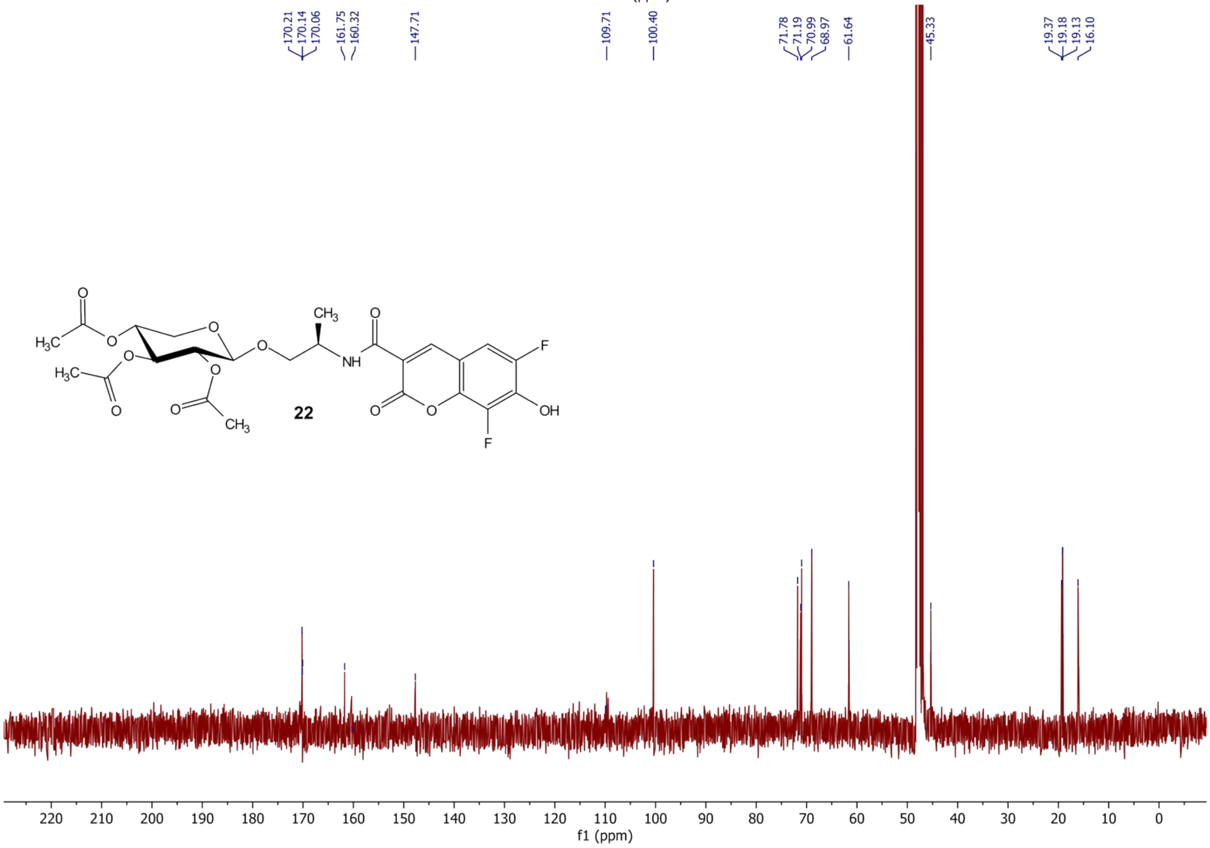

Figure S21 – ^{1}H and ^{13}C spectrum for compound **22**

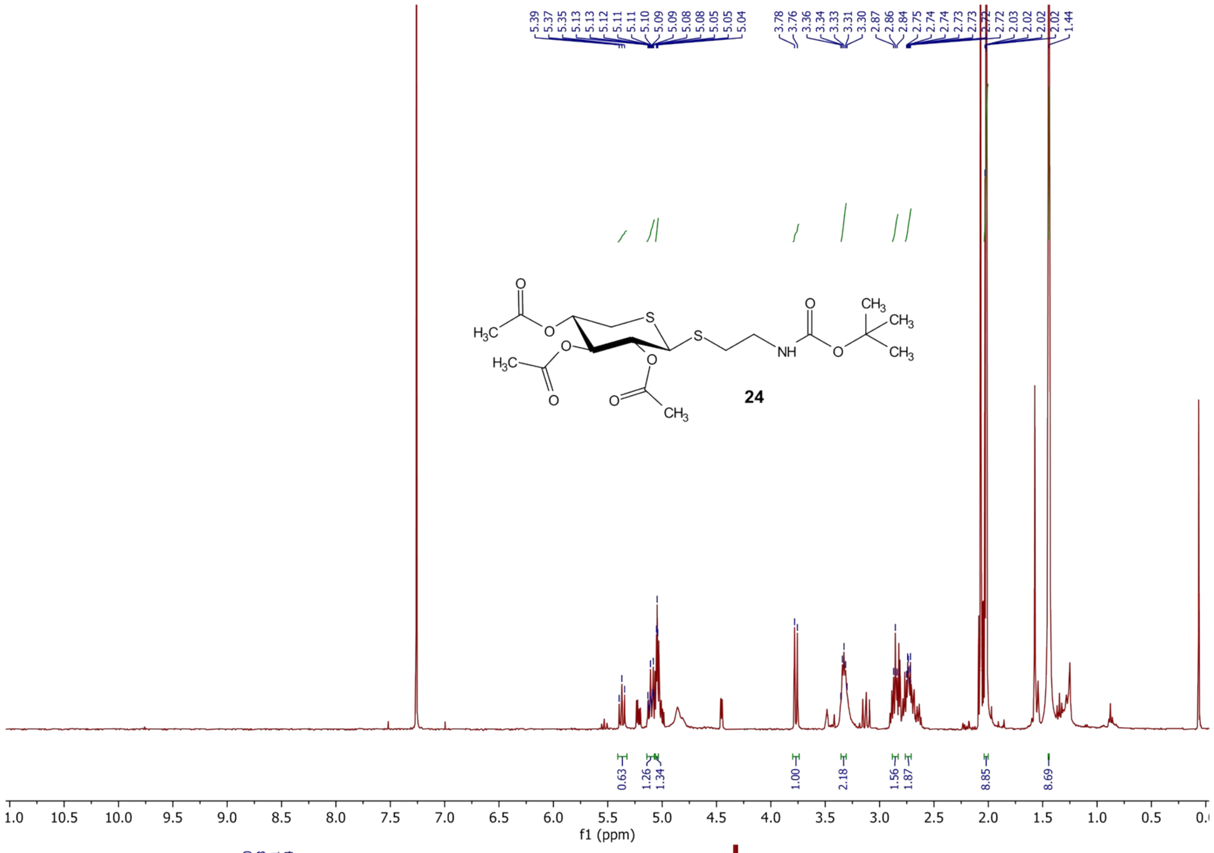

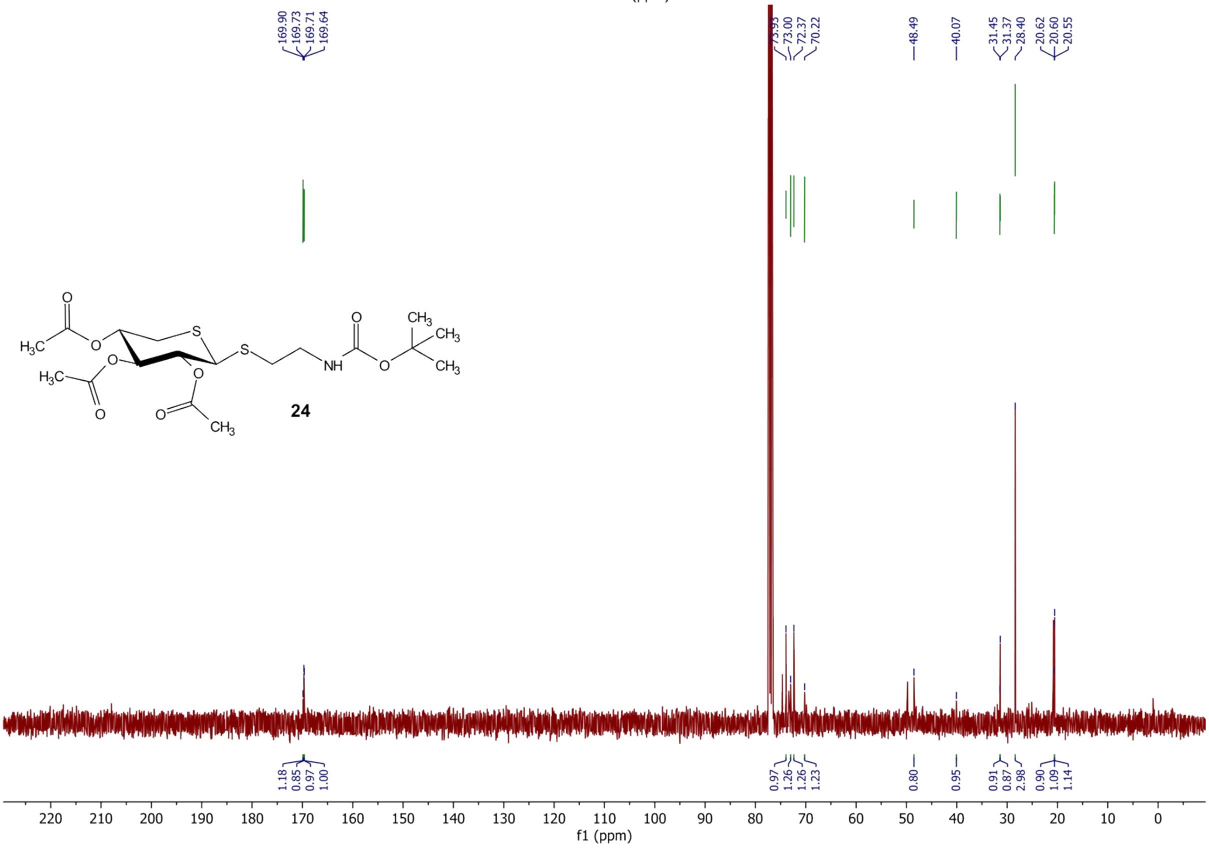

Figure S22 – ^{1}H and ^{13}C spectrum for compound **24**

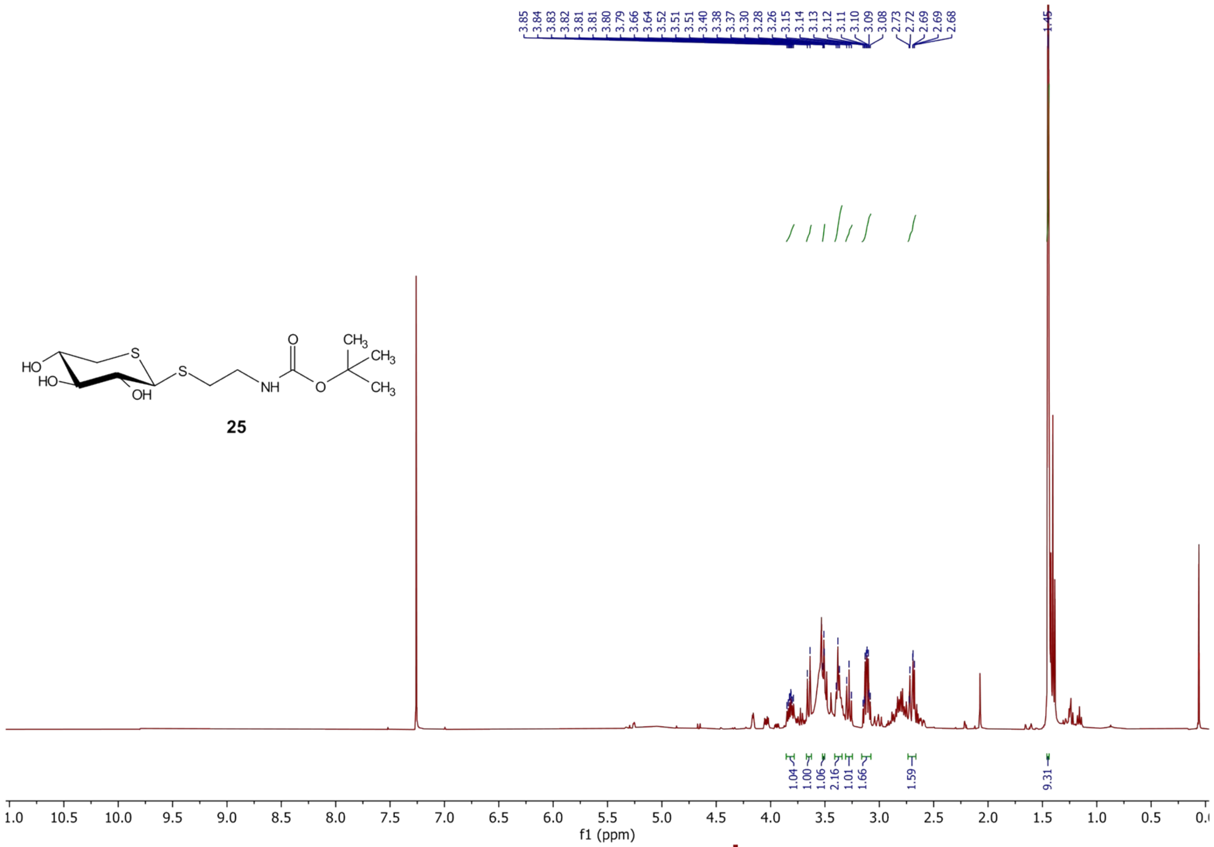

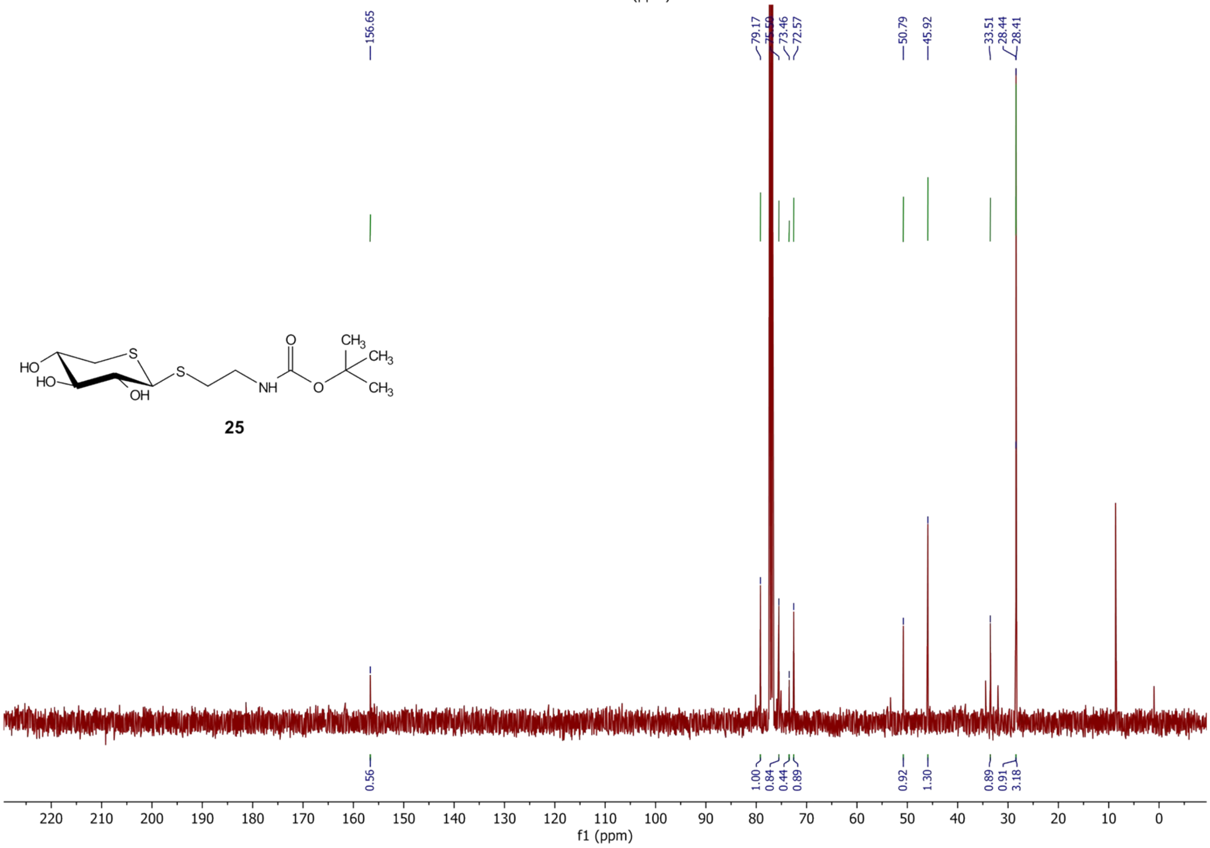

Figure S23 – ^{1}H and ^{13}C spectrum for compound **25**

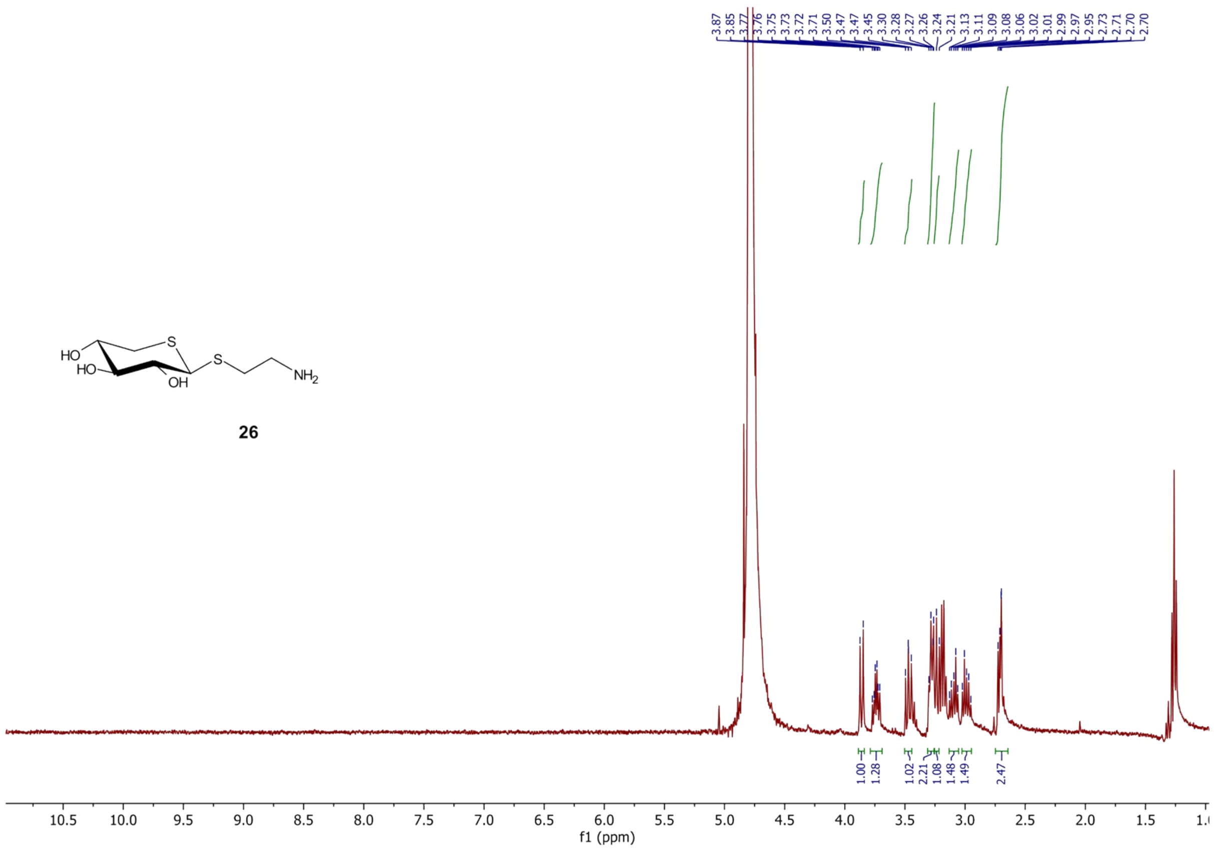

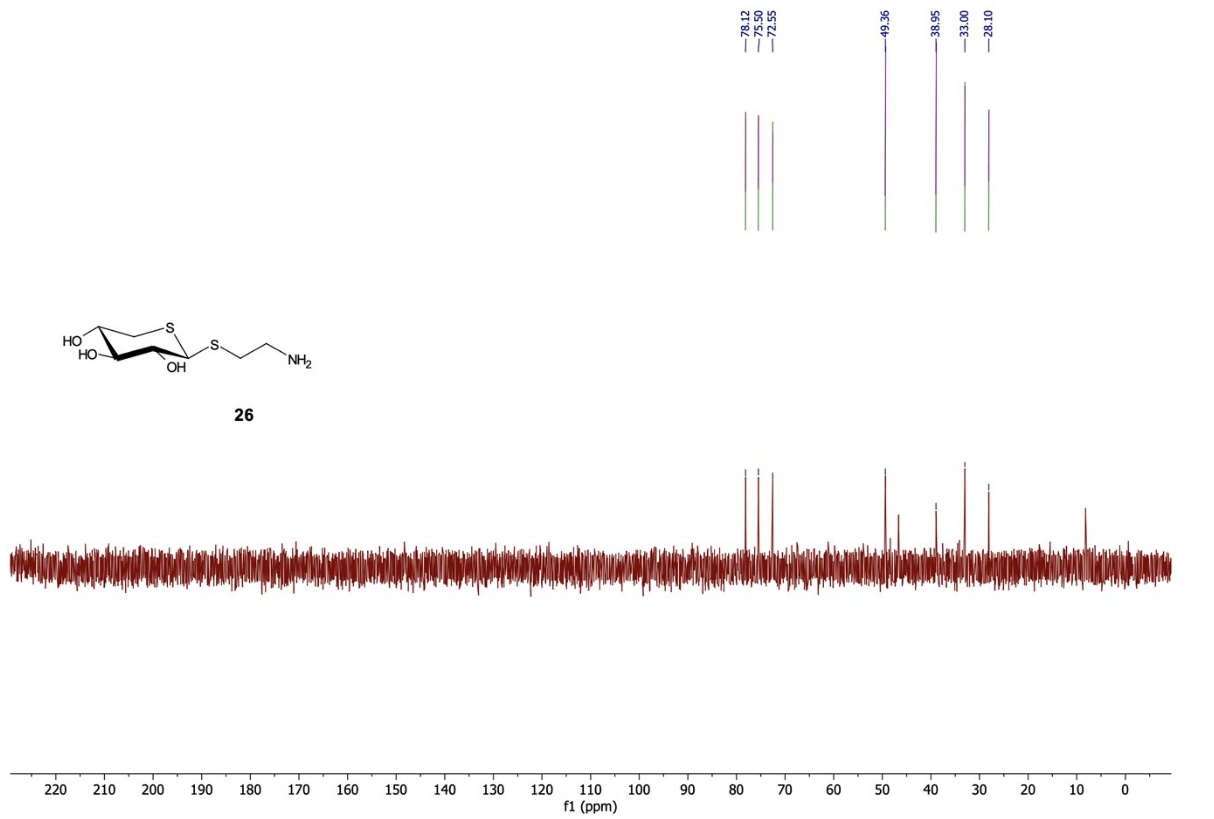

Figure S24 – ^{1}H and ^{13}C spectrum for compound **26**
